# Nanotechnology based gas delivery system: a “green” strategy for cancer diagnosis and treatment

**DOI:** 10.7150/thno.98884

**Published:** 2024-08-26

**Authors:** Meixu Chen, Tianyue Xu, Linlin Song, Ting Sun, Zihan Xu, Yujie Zhao, Peixin Du, Ling Xiong, Zhankun Yang, Jing Jing, Hubing Shi

**Affiliations:** 1 Institute of Breast Health Medicine, State Key Laboratory of Biotherapy, West China Hospital, Sichuan University and Collaborative Innovation Center, Chengdu, Sichuan, China, 610041.; 2 Department of Ultrasound & Laboratory of Ultrasound Medicine, West China Hospital, Sichuan University, Chengdu, Sichuan, China, 610041.; 3 Department of Critical Care Medicine, West China Hospital, Sichuan University, Chengdu, Sichuan, China, 610041.; 4 College of Chemical Engineering, Shijiazhuang University, Shijiazhuang, Hebei, China, 050035.

**Keywords:** Gas therapy, Delivery systems, Controlled release, Cancer treatment, Nanomedicine

## Abstract

Gas therapy, a burgeoning clinical treatment modality, has garnered widespread attention to treat a variety of pathologies in recent years. The advent of nanoscale gas drug therapy represents a novel therapeutic strategy, particularly demonstrating immense potential in the realm of oncology. This comprehensive review navigates the landscape of gases endowed with anti-cancer properties, including hydrogen (H_2_), carbon monoxide (CO), carbon dioxide (CO_2_), nitric oxide (NO), oxygen (O_2_), sulfur dioxide (SO_2_), hydrogen sulfide (H_2_S), ozone (O_3_), and heavier gases. The selection of optimal delivery vectors is also scrutinized in this review to ensure the efficacy of gaseous agents. The paper highlights the importance of engineering stimulus-responsive delivery systems that enable precise and targeted gas release, thereby augmenting the therapeutic efficiency of gas therapy. Additionally, the review examines the synergistic potential of integrating gas therapy with conventional treatments such as starvation therapy, ultrasound (US) therapy, chemotherapy, radiotherapy (RT), and photodynamic therapy (PDT). It also discusses the burgeoning role of advanced multimodal and US imaging in enhancing the precision of gas therapy applications. The insights presented are pivotal in the strategic development of nanomedicine platforms designed for the site-specific delivery of therapeutic gases, heralding a new era in cancer therapeutics.

## 1. Introduction

Cancer, characterized by its profound heterogeneity and complexity, has emerged as a significant threat to global health 1. In the face of this challenge, the medical community has been tirelessly exploring innovative therapeutic strategies. Traditional treatment modalities, including surgery, RT, and chemotherapy, have demonstrated some efficacy in clinical settings 2. However, their side effects, potential to induce drug resistance, and the promotion of tumor metastasis and recurrence have limited their therapeutic effectiveness.

As the understanding of cancer has deepened, the importance of the tumor microenvironment (TME) has become increasingly recognized. The TME exhibits unique pathological features, such as hypoxia, high reducibility, mild acidity, overexpression of H_2_O_2_, and enhanced vascular permeability—largely a result of the rapid metabolic activity of cancer cells 3. These characteristics provide the conditions necessary for tumor growth, proliferation, drug resistance, and metastasis. While interventions targeting certain TME factors may trigger compensatory self-repair or resistance, the disruption of signaling pathways can effectively dismantle the TME.

The discovery and development of NO as the first biomedical gas, due to its significant role in the treatment of cardiovascular diseases, was awarded the Nobel Prize in Physiology or Medicine in 1998 4. Since then, other therapeutic gas molecules, including NO, H_2_, CO, O_2_, H_2_S, SO_2_, and O_3_, have been identified for their potential in biomedical applications, particularly in cancer therapy. Within the TME, endogenous gasotransmitters such as NO, CO, and H_2_S play crucial roles in the growth, proliferation, and metastasis of cancer cells 5. At low concentrations, these gasotransmitters can protect cancer cells through antioxidant, signaling, and bioenergetic mechanisms, thereby facilitating tumor growth and metastasis. Conversely, at high concentrations, they are toxic to cancer cells by inhibiting mitochondrial respiration. Precise control of the concentration of these gasotransmitters is essential for cancer therapy 6. And the key concentration parameters and cancer therapeutic mechanisms of gases are shown in **Table 1**.

Compared to NO, CO, and H_2_S, H_2_ is a safer anti-cancer gas, as it does not pose the risk of blood poisoning even at high concentrations and does not promote tumor growth 7. O_2_ and O_3_ have distinct mechanisms of action in cancer therapy. The delivery of O_2_ within tumors primarily aims to alleviate hypoxia in the TME, thereby preventing tumor angiogenesis and metastasis and enhancing the effectiveness of other oxygen-dependent therapies 8. O_3_ primarily increases the levels of reactive oxygen species (ROS) within the TME and cells, enhancing the toxic effects on cancer cells. Additionally, NO and SO_2_ can cause oxidative damage to various cellular organelles of cancer cells and deplete the overexpressed glutathione (GSH) in the TME, leading to oxidative stress 9.

Most therapeutic gases not only enhance the efficacy of traditional treatment methods but also reduce their toxic side effects. Therefore, gas therapy is often used in conjunction with conventional therapies such as chemotherapy and radiotherapy. However, the uncontrolled diffusion of therapeutic gases within the body limits their effective accumulation at the target site, reducing therapeutic efficacy and potentially posing the risk of blood poisoning, especially for gases like NO, CO, and H_2_S 10. To address this issue, scientists have leveraged nanotechnology to develop nanomedicine capable of controlled gas release. These nanomedicines, by integrating gas or gas-releasing molecules (GRMs) with nanocarriers through active and passive targeting approaches, achieve precise delivery of gases to tumors. The development of stimulus-responsive GRMs and the application of multifunctional nanocarriers have enabled controlled gas release, enhancing the targeting and efficacy of gas therapy while minimizing potential side effects 11.

In recent years, the advent of nanotechnology has paved the way for precise *in vivo* gas delivery and controlled release, providing a fresh perspective for precision gas therapy. This review comprehensively discusses the mechanisms of therapeutic gas treatment, the construction of gas delivery systems, and the application of gases in cancer therapy, including imaging and combination therapies. The application of nanotechnology holds promise for gas therapy to become a more effective and less toxic approach to cancer treatment.

## 2. Historical background of gas therapy

**Figure 1:**
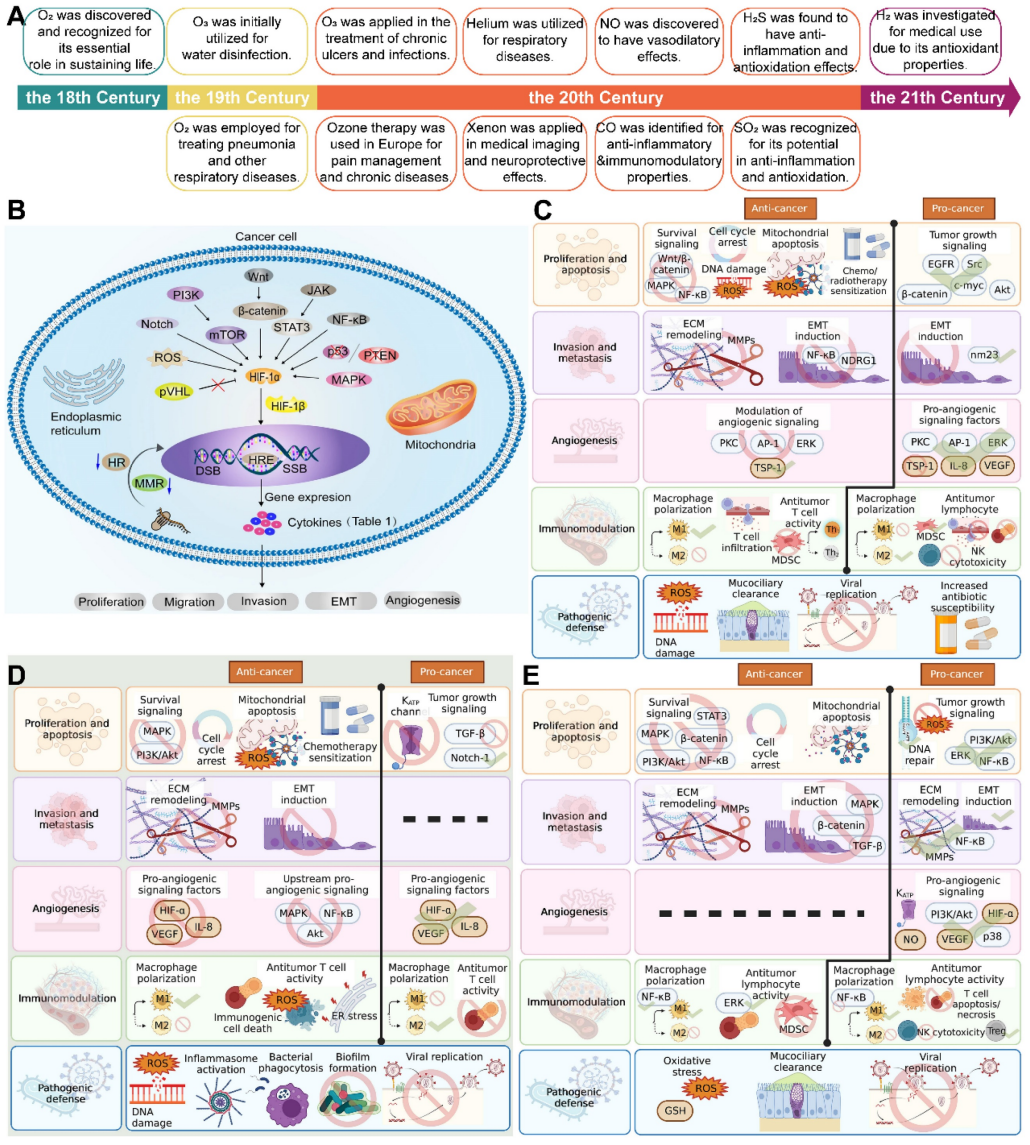
The history of gas therapy and the molecular mechanisms of major gases. (A) Advancements and key milestones in gas therapy. (B) Biological changes in cancer cells adapt to hypoxia. Adapted with permission from 24, copyright 2023 Zhou Chen *et al.* (C) NO effects in the context of cancer. Adapted with permission from 39, copyright 2023 Elsevier. (D) CO effects in the context of cancer. Adapted with permission from 39, copyright 2023 Elsevier. (E) H_2_S effects in the context of cancer. Adapted with permission from 39, copyright 2023 Elsevier.

Gas therapy, an interdisciplinary field at the confluence of medicine, chemistry, and biology, has chronicled the integration and convergence of these sciences 12. A timeline overview of the major findings and advances in gas therapy is presented in **Figure 1A**. The seminal discovery of O_2_ in the 18th century, marked the dawn of a new era in understanding the vital role of gases in sustaining life. The medical applications of O_2_ expanded in the 19th century, with its significance becoming increasingly evident in the treatment of pneumonia and other respiratory diseases.

As the 20th century unfolded, hyperbaric oxygen therapy (HBOT) emerged as an innovative treatment modality, enhancing the solubility of O_2_ in the bloodstream and thereby significantly promoting wound healing and tissue repair. HBOT has demonstrated remarkable efficacy, particularly in the treatment of diving diseases and gas gangrene. Towards the end of the century, the discovery of NO paved the way for novel approaches in the treatment of cardiovascular diseases; its vasodilatory properties established inhaled NO as an effective therapeutic for pulmonary arterial hypertension and other conditions 13. Concurrently, the advent of PDT, leveraging photosensitizers to generate ROS under specific light wavelengths, introduced new treatment options for cancer and certain skin diseases.

The 21st century has seen H_2_ being investigated for medical purposes due to its antioxidant properties, with its potential applications in reducing oxidative stress and inflammation being extensively explored 14. Helium (He), applied earlier in the 20th century, has been utilized in the treatment of severe asthma and chronic obstructive pulmonary disease (COPD), with its physical properties aiding in reducing airway resistance. The discovery of the potential therapeutic roles of CO and SO_2_ in anti-inflammation, antioxidation, and cytoprotection towards the end of the 20th century has added new dimensions to the applications of gas therapy 15.

At the close of the 19th century, German physician Wolfram von Siemens pioneered the application of O_3_ for water disinfection, ushering in its use in the medical field. By the 1950s, O_3_ had been adopted for the treatment of chronic ulcers and infections, and by the 1980s and 1990s, O_3_ therapy was widely applied in Europe for pain management and the treatment of certain chronic diseases.

Xenon (Xe), a noble gas with significant applications in medical imaging due to its high atomic number, enhancing the contrast in computed tomography (CT) and magnetic resonance imaging (MRI), has been used in medical imaging since the 1970s. Although research into its therapeutic applications is relatively new and primarily focused on its potential neuroprotective effects, the specific timeline for its use in therapy remains to be fully established 16.

The contemporary evolution of gas therapy owes much to the in-depth study of the biological effects of gases and the technological advancements in gas delivery systems, such as nanotechnology and drug delivery systems, which have made gas therapy more precise and effective. Ongoing clinical trials and research are continually validating the safety and efficacy of gas therapy. This progress, coupled with the deepening understanding of disease mechanisms and the relentless advancement of science and technology, promises the emergence of innovative therapeutic approaches in the realm of gas therapy.

## 3. Members of therapeutic gas

### 3.1. Oxygen

Deep understanding of the complex metabolic landscape of cancers has led to metabolic reprogramming as a defining characteristic of malignant progression 17. Cancer cells exhibit a remarkable ability to autonomously regulate their metabolic pathways, meeting their high demands for bioenergy and anabolic processes necessary for their unbridled proliferation and survival, while simultaneously countering the oxidative stress that accompanies such rapid growth 18. At the core of this metabolic process is the Warburg effect, which describes how cancer cells primarily use glucose to make lactate 19. This happens even when there is plenty of O_2_ around. The production of lactate helps these cells meet their energy and basic requirements 20. This metabolic strategy, while effective for the cancer, inadvertently leads to endothelial dysfunction and insufficient O_2_ delivery, owing to the excessive metabolic demands on the blood vessels. Consequently, a chronic hypoxic environment emerges, promoting the activation of hypoxia-inducible factors (HIF) and driving the malignant progression of tumor by enhancing growth, invasiveness, and metastatic potential 21.

Hypoxia, a common feature of solid tumors, arises from the inadequate supply and voracious consumption of O_2_, typically characterized by an O_2_ partial pressure below 5 mmHg 22. It has been implicated in the promotion of abnormal cell proliferation, formation of heterogeneous vascular structures, and dysfunction of lymphatic systems, all of which contribute to metastasis, angiogenesis, multidrug resistance, and resistance to RT 23. The hypoxic TME poses a significant challenge for therapies that rely on O_2_ consumption, such as PDT, which requires the conversion of oxygen into cytotoxic ROS **(Figure 1B)** 24. To address this limitations, a multitude of strategies have been proposed to mitigate tumor hypoxia, including increasing blood O_2_ levels, delivering O_2_ directly to the tumor site, and the provision of O_2_ through the catalytic decomposition of endogenous substances (such as H_2_O_2_) or illuminating photosynthetic bacteria to provide O_2_ 25. Hyperbaric oxygen therapy has been employed to enhance systemic O_2_ levels, serving as an adjunct to cancer therapy 26. For the effective delivery of O_2_, micro- and nano-carriers have been engineered to encapsulate free O_2_ molecules or oxygen-generating catalysts, which can be activated by specific stimuli such as near-infrared laser (NIR) radiation, US, or X-rays 27. Additionally, strategies leveraging the hypoxic characteristics of tumors for drug release while concurrently alleviating hypoxia have been employed 28. Despite these advances, challenges remain, including the complexity of preparation, limited oxygen loading efficiency, premature leakage, and suboptimal tumor targeting.

Future research must delve deeper into the efficacious delivery of O_2_ to tumor tissues while minimizing damage to healthy tissues. The development of materials with high O_2_ affinity, such as hemoglobin (Hb), perfluorocarbon (PFC) 29, and metal-organic frameworks (MOFs) 30, may offer novel solutions for O_2_ transportation. Innovative nanotechnologies, like camouflaging nanoparticles with red blood cell membranes, hold promise for enhancing the tumor targeting and therapeutic efficacy of O_2_ carriers 31. O_2_-based therapeutic strategies are anticipated to be integrated with current cancer treatment modalities, forming a multimodal approach to treatment. By precisely controlling the release and delivery of oxygen, the TME can be finely modulated, thereby improving therapeutic outcomes and reducing side effects. With the continuous advancement in fields such as materials science, nanotechnology, and biomedical engineering, there is ample reason to believe that O_2_ therapy will play an increasingly pivotal role in the field of oncology.

### 3.2. Nitric oxide

NO is the first gas molecule discovered to participate in the complex transduction of cell signal transduction 32, It plays a key role in physiological regulation, such as cardiovascular system regulation 33, immune regulation 34, and neurotransmission 35. The synthesis of intracellular NO is mainly through the conversion of L-arginine into L-citrulline by nitric oxide synthase (NOS), which ultimately produces the multifunctional molecule 36. NO wields a dual-edged sword in the biological realm, the effects oscillating between protective and deleterious, depending on its concentration. At low physiological levels, NO serves as an antioxidant, fostering tumorigenesis by mitigating the Fenton reaction, quelling free radical chain reactions, and curbing the enzymatic activities of peroxidases and oxidases 6. In stark contrast, when NO is present in surplus, it induces cancer cell apoptosis by a variety of mechanisms to exert anti-cancer effects in the body. These mechanisms include the upregulation of the p53 gene 37, the amplification of cytochrome C release from mitochondria, protein nitration, and the formation of cytotoxic peroxynitrite (ONOO^-^). However, an overabundance of NO can precipitate in neurotoxicity, disruption of cellular homeostasis, and alter protein function, potentially culminating in genetic mutations and oncogenesis 38. The mechanism diagram of NO promote and against cancer is shown in **Figure 1C** 39.

The physiological nuances of NO have ignited a surge of preclinical research, particularly in cancer treatment. Nonetheless, the propensity of NO to diffuse rapidly and its transient presence in plasma results in insufficient tumor accumulation and suboptimal therapeutic efficiency 40. These challenges have catalyzed an upsurge in research on NO donors and their delivery systems, with the ultimate goal of controlled gas release in response to endogenous or exogenous stimuli and ensuring accumulation at tumor sites via passive or active targeting strategies. A series of structurally diverse NO donors, including organic nitrates/nitrites to metal-NO complexes, nitrosamines, S-nitrosothiols, diazeniumdiolates, and sydnonimines, each with distinct chemical reactivities and release kinetics, have emerged as promising candidates in the arena of biological research, offering a lighthouse of hope for the development of novel therapeutic modalities.

Looking ahead, research on NO for cancer treatment is likely to focus on several key areas: First, the development of novel NO donors with improved chemical stability and controllable release profiles to enhance therapeutic efficacy and minimize side effects. Second, the investigation and optimization of NO delivery systems, employing passive or active targeting strategies to achieve specific accumulation of NO in tumor tissues. Furthermore, by integrating modern nanotechnology, intelligent NO release systems can be designed to respond to specific biomarkers, thereby improving the precision and efficiency of treatment. Additionally, considering the dual role of NO, future research will also delve into its mechanisms of action in different types of cancers and stages, aiming to realize more personalized therapeutic strategies. Through these efforts, the application of NO in cancer treatment will continue to make breakthroughs, bringing new hope and more effective treatment options to cancer patients.

### 3.3. Carbon monoxide

CO, once regarded as a noxious atmospheric pollutant, emerges from the incomplete combustion of organic substances. It is known for its strong affinity for Hb, potentially diminishing blood's oxygen-carrying capacity by usurping the oxygen-binding sites on Hb 41. CO serves as a signaling molecule within the neuronal system, where it is implicated in the modulation of neurotransmitter and neuropeptide release, thereby influencing processes such as learning, memory, and olfactory adaptation. Additionally, CO exerts vasorelaxant effects and confers cardio-protection, playing a pivotal role in the immune, respiratory, reproductive, gastrointestinal, renal, and hepatic systems 42. CO exerts its physiological effects by binding to various transition metal-containing enzymes and ion channels, including soluble guanylate cyclase and cytochrome oxidase. This interaction modulates the cellular response to CO. Furthermore, CO activates NOS, leading to an increase in NO production, which in turn affects vascular relaxation and blood pressure regulation. In terms of signal transduction, CO primarily elevates cyclic guanosine monophosphate (cGMP) levels by activating soluble guanylate cyclase (sGC), a process that involves the binding of CO to the ferrous heme iron (Fe^2+^) 43.

The metabolic pathway of CO involves the degradation of heme, yielding CO, iron ions, and biliverdin, which is rapidly reduced to bilirubin. Endogenous CO freely traverses cellular membranes, thereby mediating alterations in cellular function. However, excessive inhalation of CO can lead to poisoning, with mechanisms including the formation of carboxyhemoglobin (COHb) that induces tissue hypoxia, the generation of ROS through oxidative stress leading to cellular damage, and the disruption of CO signaling systems that affect vascular relaxation and platelet aggregation, among other physiological functions. Clinically, the enhancement of endogenous CO production or the direct administration of exogenous CO has been applied in various therapeutic areas, such as anti-inflammatory, anti-apoptotic, and anti-proliferative treatments for smooth muscle. The level of COHb serves as a significant biomarker for the assessment of CO levels in the human body. Nevertheless, the threshold distinguishing the physiological effects from toxic effects of CO is not well-defined and is influenced by the concentration and duration of CO exposure.

The exploration of physiological roles of CO beyond its Hb-binding capacity opens up new avenues for cancer therapeutics. Similar to NO, the future trajectory of CO may pivot around the creation of innovative CO-releasing molecules (CO-RMs). CO-RMs, characterized predominantly by their stimulus-responsive nature, encompass a range of activation modalities such as photoactivation, sonoactivation, chemical triggering, and bioorthogonal click chemistry. The substantial potential of these stimuli in activating CO-RMs offers a more precise control over spatiotemporal dynamics and dosage compared to endogenous stimuli. Potential mechanisms of action of CO delivered as a gas is shown in **Figure 1D** 39. While CO-RMs offer new horizons for the clinical application of CO, the development and application of these molecules are not without challenges. Ensuring the stability of CO-RMs is a critical issue, as is enhancing the specificity of CO release in terms of both time and space. Additionally, managing the potential toxicity of CO-RMs is another challenge that must be addressed to facilitate their safe and effective clinical use.

### 3.4. Hydrogen sulfide

In the field of gasotransmitters, H_2_S is the third member of this biologically significant group, following in the footsteps of CO and NO. H_2_S is applied in a diverse array of physiological processes and has been explored for its therapeutic potential in a range of diseases, including Alzheimer's disease, diabetes, and cancer 44. Endogenous H_2_S is mainly produced by cystathionine-β-synthase (CBS), cystathionine γ-lyase (CSE), and 3-mercaptopyruvate sulfurtransferase (3-MST), as well as by the microbial flora residing in the gastrointestinal tract 45. The effects of in the context of cancer is shown in **Figure 1E** 39. Similar to NO, H_2_S exhibits bidirectional effects in the regulation of cell proliferation and death, with its effects depending on concentration and whether it is exposed to reactive sulfur species (RSS). At physiological concentrations (in the micromolar range), H_2_S has been shown to promote neovascularization around tumor sites by activating K^+^-ATP channels and the sGC-cGMP signaling cascade 46. Conversely, at elevated concentrations (in the millimolar range), H_2_S displays its cytotoxicity by inhibiting mitochondrial function, triggering oxidative stress and apoptosis 47.

H_2_S donors are categorized into inorganic, such as H_2_S itself and sodium sulfide (Na_2_S), and organic forms. Inorganic donors face challenges due to rapid oxidation in water, affecting H_2_S release uniformity and therapeutic consistency. Organic donors, including ADT-OH, thiobenzamide, GYY4137, DADS, and DATS, offer a more controlled H_2_S production. Pharmaceutical H_2_S donors like ATB-346 and GIC-1001 are in Phase II trials, highlighting advancements in H_2_S therapy. Nanotechnology-based donors provide precise H_2_S release, optimizing efficacy and safety. Stimuli-responsive donors, sensitive to pH, light, or free radicals, are innovative tools tailored for specific physiological or pathological triggers, showcasing the adaptability of H_2_S therapeutics 45.

The exploration of H_2_S in tumor genesis and the therapeutic implications necessitates a cautious and innovative approach. Strikingly, colon cancer cells demonstrate an increased expression of CBS, thereby augmenting the synthesis and release of H_2_S into the TME, promoting cell proliferation and angiogenesis within a concentration range of 0.3 to 3.4 mM 5, 48. H_2_S has thus been considered a therapeutic target for colon cancer. The development of sophisticated delivery systems that can modulate H_2_S levels within the TME will be paramount. Such systems would not only maximize the cytotoxic effects on cancer cells, but also minimize the exposure and subsequent adverse effects on healthy cells. Additionally, the integration of nanotechnology holds promise for the targeted delivery and controlled release of H_2_S, thereby improving the therapeutic index. As our understanding of the intricate mechanisms by which H_2_S exerts its effects in different cancer types and stages deepens, personalized treatment strategies will be devised.

### 3.5. Sulfur dioxide

SO_2_, historically regarded as an atmospheric pollutant, has emerged as a subject of intrigue in the field of physiological research. The inhalation of SO_2_ at elevated concentrations probably cause oxidative stress, precipitating damage to critical biomacromolecules, including proteins, lipids, and DNA 49. Furthermore, SO_2_ is also related to the regulation of cell membrane fluidity and the decrease of enzymatic activities, especially superoxide dismutase (SOD) and glutathione peroxidase (GPx) 50. These physiological insights have led to the recognition of SO_2_ as a significant player in various pathological conditions, such as cardiovascular diseases 9, disinfection processes, and cancer therapy 51, thus earning its place alongside NO, CO, and H_2_S as an endogenous gas signaling molecule.

Endogenous SO_2_, presents at concentrations ranging from 0 to 5 μM within living cells, is generated in the mitochondria through the catalytic action of thiosulfate sulfur transferase (TST), which facilitates the conversion of GSH and sodium sulfite (Na_2_S_2_O_3_). Additionally, sulfur-containing amino acids, such as L-cysteine, can also be converted into sulfites catalyzed by cysteine dioxygenase and glutamate-oxaloacetic aminotransferase (GOT) 52. H_2_S can also contribute to sulfite formation via enzymatic or non-enzymatic oxidative pathways, leading to the generation of SO_3_^2-^, HSO_3_^-^ and SO_2_, which collectively maintain a delicate equilibrium within the cellular milieu. However, an overabundance of these compounds can trigger oxidative stress and has been linked to age-related diseases, including rheumatoid arthritis and Parkinson's disease.

SO_2_ has been shown to interact with the thiol groups (-SH) of proteins and disulfide bonds (-S-S-), which play pivotal roles in a variety of physiological processes, such as vasodilation, inhibition of vascular smooth muscle proliferation, and modulation of cardiac function. Recent investigations have revealed the therapeutic potential of SO_2_ in cancer treatment by overcoming drug resistance and maintaining cellular homeostasis 53. Future research endeavors must address several challenges associated with the application of SO_2_, including its short half-life 54, low stability, and limited biocompatibility.

Potential avenues of investigation encompass the development of novel SO_2_ donors or prodrugs capable of stably releasing SO_2_ within the body, with enhanced targeting and bioavailability. SO_2_ donors constitute a class of compounds capable of releasing SO_2_ in biological systems. This group encompasses a variety of chemical entities, including inorganic sulfates and sulfites, organic sulfonates and sulfites, sulfinic acids, metal-sulfur complexes, polymeric SO_2_ donors, small molecule prodrugs, photosensitizers, enzymatic substrates, and nanocarrier systems 55. These entities facilitate the controlled release of SO_2_ through diverse mechanisms and triggering conditions. Zhang and colleagues have engineered a thermosensitive hydrogel system incorporating the SO_2_ prodrug benzothiazole sulfinate (BTS), which facilitates the release of SO_2_ under near-infrared irradiation to augment the efficacy of cancer photodynamic therapy and inhibit tumor recurrence 56. Similarly, Chen and colleagues have integrated the SO_2_ prodrug BTS with copper single-atom nanozymes (Cu SAZ) encapsulated within platelet membrane vesicles (PV). Capitalizing on the slightly acidic nature of the TME, SO_2_ is released to synergistically enhance the inhibition of gastric cancer ascites by Cu SAZ in an efficient manner 57. As our understanding of the physiological and pathological mechanisms of SO_2_ deepens, coupled with the development of innovative delivery systems, precise control over the release and action site of SO_2_ can be achieved. This approach aims to maximize therapeutic efficacy while minimizing potential side effects, offering new treatment options for cancer patients.

### 3.6. Hydrogen

H_2_, the most diminutive of gases, has garnered attention for its regulatory roles in physiological and pathological processes in recent years 58. The therapeutic potential of H_2_ was initially reported in 1975, and since then, it has been the subject of extensive investigation as a novel treatment modality 59. Early medical applications of H_2_ are primarily directed towards squamous cell carcinoma of the skin, where it exhibits notable anti-cancer effects 59. Recent research has unveiled a variety of therapeutic pathways for H_2_, such as its ability to counteract oxidative stress by neutralizing potent ROS, including hydroxyl radicals (•OH) and ONOO^-^; its capacity to suppress inflammatory responses by reducing the levels of TNF-α, IL-1β, and IL-6; and its involvement in cellular survival, proliferation, and apoptosis processes through the modulation of signaling pathways like MAPK, PI3K/Akt. As a therapeutic gas, H_2_ has demonstrated promise across a range of medical applications, including inflammation, brain injury and Alzheimer's disease treatment 60.

At low concentrations, H_2_ has been observed to modulate inflammation, whereas at higher levels, it can disrupt mitochondrial respiration and redox homeostasis, accelerating cellular injury and apoptosis 61. This duality is thought to stem from the capacity of H_2_ to inhibit energy supply in cancer cells, reduce the intra-tumoral expression of vascular endothelial growth factor (VEGF), and trigger a systemic immune response 62. Delivery methods for H_2_ include inhalation of H_2_-enriched air 63, administration of H_2_-rich saline injections 64, and oral intake of H_2_-rich water 65. Clinical trials have explored the therapeutic benefits of H_2_-rich water and saline in conditions related to oxidative stress and inflammation, such as type II diabetes, metabolic syndrome, stroke, Parkinson's disease, hypercholesterolemia, and colorectal cancer 58.

However, the low water solubility and profound tissue penetration ability of H_2_ present challenges in achieving controlled release and targeted delivery. Nanomedicine strategies have emerged as a modality for H_2_ treatment, facilitating direct delivery or stimuli-responsive release 58. For instance, the reaction between reducing metals, such as magnesium (Mg) powder, and water to generate H_2_ has been applied for the treatment of osteoarthritis and other inflammation-related disorders 66. Additionally, implantable materials and devices incorporating H_2_-based cancer treatment have been applied in liver and prostate cancer therapy, yielding successful outcomes with minimal systemic toxicity 67. The reaction between Mg and water, however, is relatively slow, potentially leading to inadequate H_2_ production for gas-based anti- cancer therapy. To enhance local H_2_ saturation and tumor targeting, nanoparticles, including iron (Fe), palladium hydride (PdH_0.2_), and magnesium diboride (MgB_2_), have been utilized as H_2_ production carriers 68. It is imperative to consider the degradability and cytotoxicity of these nanoparticles, as they could pose additional health risks. Advances in these nanocarriers are anticipated to improve the delivery efficiency and efficacy of H_2_, thus expanding its therapeutic applications.

### 3.7. Ozone

O_3_, recognized for its potent oxidizing properties, has been a staple in sewage treatment for its effectiveness. In the medical field, particularly in Europe, O_3_ therapy has been utilized for decades, showing its versatility in treating a range of conditions, including cardiovascular and cerebrovascular diseases 69, wound healing 70, skin diseases 71 and rheumatoid diseases 72. The interaction of O_3_ with hydrogen atoms, as depicted in equations (1) to (5), enhances irradiation efficiency by transforming hydrogen atoms into •OH, thereby augmenting the therapeutic impact of ionizing irradiation. As a secondary outcome, O_3_ can alleviate hypoxia within the TME, subsequently improving the efficacy of RT 73.

Equations (1) to (5) illustrate the chemical mechanisms by which O_3_ participates in these processes:

H+O_3_ → HO_3_ Equation (1) 74

HO_3_• ↔ H^+^ + O_3_•^-^ Equation (2) 75

e^-^_aq_ + O_3_ → O_3_•^-^ Equation (3) 75

O_3_•^-^ + H^+^ → •OH + O_2_ Equation (4) 75

H_2_O_2_ +O_3_ → •OH + O_2_ Equation (5) 76

At high concentrations, O_3_ is not only cytotoxic but also prompts the overproduction of ROS, which can lead to endoplasmic reticulum stress (ERS), apoptosis, and the release of danger-associated molecular patterns (DAMPs) such as calreticulin (CRT), ATP, and HMGB1. The release of these DAMPs can initiate immune responses and foster immunogenic cell death (ICD) of cancer cells 77. Research has indicated that O_3_ therapy, when combined with other treatments, can significantly enhance anti-cancer effects and mitigate the adverse effects of chemotherapy through its radioprotective properties 78.

Despite the substantial potential of O_3_ in cancer therapy, achieving precise delivery and controlled release remains a key challenge. Prior investigations have established a safe and effective O_3_ liposome delivery system that synergize with RT, and has been shown to alter the immunosuppressive TME of triple-negative breast cancer (TNBC) and enhance the anti-cancer effects of PD-1 monoclonal antibody treatment 79. Song *et al.*, integrated poly(lactic-co-glycolic acid) (PLGA) with iRGD and O_3_-containing PFC, enabling targeted O_3_ delivery to tumors. This system leveraged microwave (MW) irradiation to trigger the release of O_3_ and induce cancer cell death 80. Given that the components utilized in these O_3_ carriers are clinically approved and deemed safe, O_3_-based cancer treatments are expected to become a promising clinical application, pending further optimization and validation.

## 4. Gas carriers for direct delivery

### 4.1. Perfluorocarbons

Perfluorocarbons (PFCs), often referred to as “gas-like liquids” or “gases in liquid form”, are distinguished by their low polarity and weak intermolecular forces, a consequence of high electronegativity of fluorine and compact atomic radius 81. These properties endow PFCs with the unique ability to dissolve gases via a mechanism known as “similar phase dissolution”. The weak intermolecular forces further permit gases to readily permeate the interstitial spaces among PFCs molecules. Perfluorodecalin (PFD, C_10_F_18_), perfluorohexane (PFH, C_6_F_14_), and perfluoropentane (PFP, C_5_F_12_) are commonly employed among the PFCs, boasting a robust gas-binding capacity for gases such as O_2_, CO_2_, and H_2_, with affinities significantly exceeding that of water 82. A compilation of the physical properties of these PFCs is presented in **Table 2.** The entrapped gases within PFCs can be induced to release upon exposure to specific stimuli, such as heat, US, or light, rendering PFCs a popular choice as gas carriers in preclinical research 83.

In the context of cancer treatment, PFCs have been harnessed as gas carriers in preclinical studies, integrated into nanostructures to enhance the efficacy of PDT 84, sonodynamic therapy (SDT) 85, RT 86, and to counteract tumor immunosuppression by mitigating hypoxia within the TME 87. However, the challenge of potential O_2_ leakage during the delivery process and the reliance on concentration gradients for O_2_ diffusion, which complicates the maintenance of elevated O_2_ levels across multiple treatments, still remain pressing issues 88.

**Figure 2:**
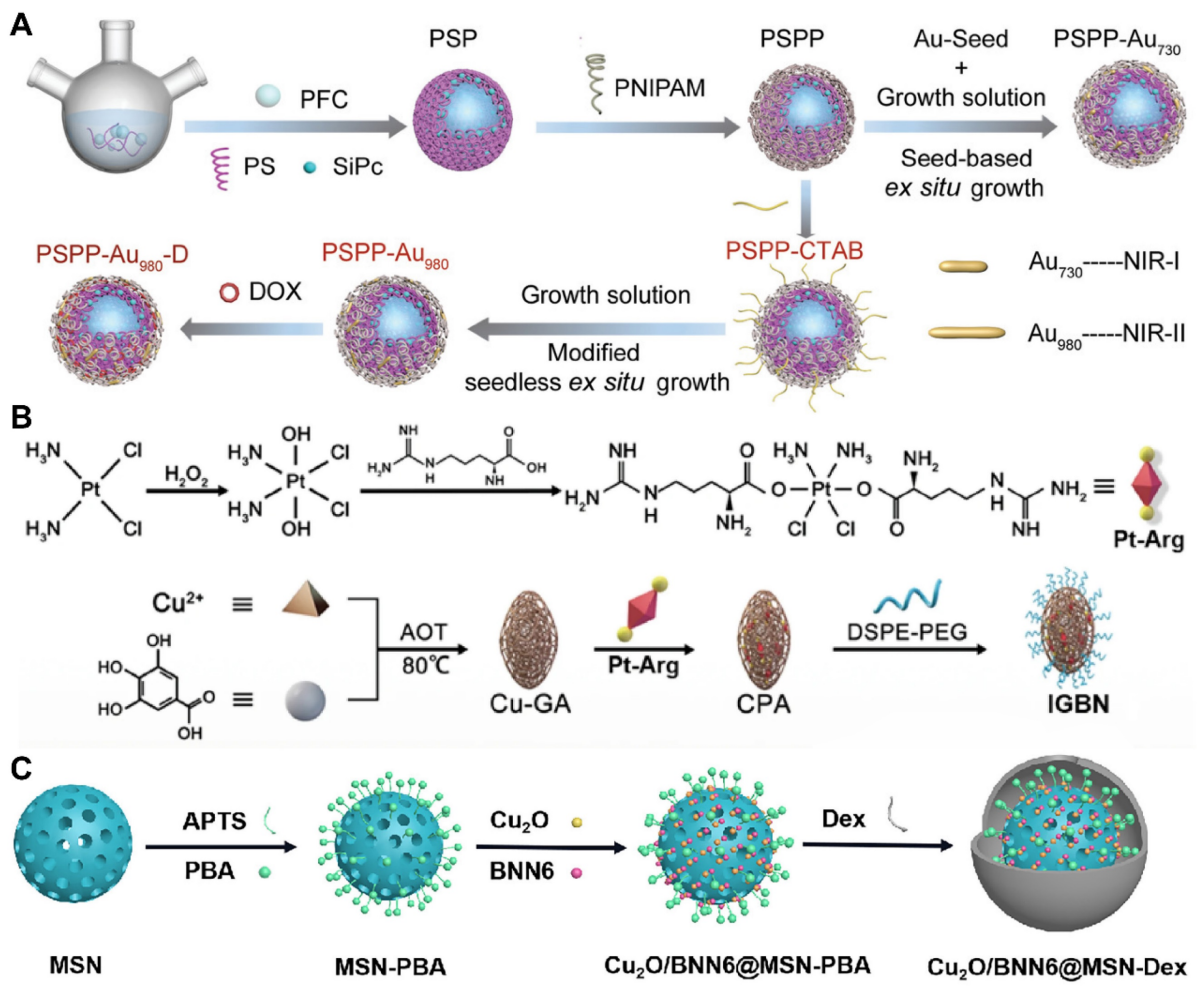
Gas delivery system using PFC, MOF and silica-based nanoplatform as gas carriers. (A) An oxygen carrier (PSPP-Au_980_-D using PFC). Adapted with permission from 89, copyright 2022 Wiley-VCH. (B) A copper-based MOF loaded with cisplatin-arginine (Pt-Arg) prodrug. Adapted with permission from 96, copyright 2022 Sijie Wang *et al.* (C) A silica-based nanoplatform (Cu_2_O/BNN6@MSN-Dex). Adapted with permission from 100, copyright 2023 Elsevier.

To tackle these challenges, researchers have engineered controllable gas carriers and stimuli-responsive systems for PFCs-based cancer therapy. Zhang* et al.* developed a NIR-II photothermal-responsive “oxygen bomb”, designated as PSPP-Au980-D, which released O_2_ into the anoxic TME under a 980 nm laser, simultaneously triggering local congestion and doxorubicin (DOX) release, followed by the application of a 680 nm laser to generate singlet oxygen (^1^O_2_) for synergistic PDT and chemotherapy **(Figure 2A)** 89. Future studies ought to concentrate on improving the compatibility of PFCs with biological systems and ensuring their enduring safety by conducting thorough preclinical examinations. The development of advanced delivery systems that allow for controlled release of the payload in response to specific biological cues or stimuli is also essential. Such systems would not only improve the therapeutic index but also minimize the potential for adverse effects. Furthermore, understanding the pharmacokinetics of PFCs, including their metabolism, distribution, and excretion, is critical for optimizing dosing regimens and ensuring patient safety. With these advancements, PFCs have the potential to become a transformative platform for gas delivery in various therapeutic applications.

### 4.2. Metal-organic frameworks

MOFs have garnered immense interest due to their strategic assembly through the coordination of metal ions or clusters with organic linkers. Characterized by their exceptionally high porosity, customizable structures, and tunable functionalities, these crystalline materials have been extensively utilized in applications such as solar energy conversion, photocatalysis, molecular sensing, gas storage, and drug delivery 90. Over 20,000 MOFs have been synthesized to date, with variants like MIL-53, HKUST-1, and Fe-BTC being commercialized 91.

MOFs exhibit a broad potential for application in gas storage and separation, owing to their porosity and structural malleability. For instance, the HKUST-1 can adsorb up to 267 cm³ (STP)/cm³ of methane at room temperature and 6.5 MPa pressure 92, nickel-based MOFs can store up to 23.0 g/L of H_2_ between -75°C and 25°C 93, and cadmium-based MOFs have also demonstrated high storage capacity for acetylene. Furthermore, MOFs have been employed in the capture and separation of CO_2_ and SO_2_, as well as the capture of toxic gases in industrial exhaust, effectively achieving separation between acetylene and CO_2_, and between olefins and paraffins 94.

However, the application of MOFs as gas carriers faces certain limitations. Firstly, some metal ions may be toxic to living organisms, and the biodegradability of organic ligands can also affect the safety of MOFs 95. Secondly, the complex synthesis process of MOFs limits their feasibility for large-scale applications. To overcome these challenges, a multitude of enhancements must be pursued. For example, developing simple synthetic strategies and methods to enhance the synthesis efficiency and reduce costs of MOFs; improving the stability in human bodies and biocompatibility of MOFs through material design and post-modification; and utilizing computer simulations and visual synthetic equipment to aid in the synthesis process of MOFs to improve repeatability and controllability.

Despite the significant advantages of MOFs in gas storage and separation, there is a relative scarcity of literature on the direct use of MOFs for gas therapy. In contrast, a substantial body of work has explored the use of MOFs for the delivery of gas-prodrugs, suggesting that there may still be limitations and room for improvement in this direction. Wang *et al.* designed a copper-based MOF that delivers a cisplatin-arginine (Pt-Arg) prodrug as a NO donor to tumors. This MOF generated copper ions and degraded estrogen, while the prodrug was activated to cisplatin, increasing H_2_O_2_ levels. The designed MOF nano-system synergized copper ion effects, NO therapy, and chemotherapy for effective cancer treatment **(Figure 2B)** 96. Nevertheless, future research endeavors should not only concentrate on the synthesis and stability enhancement of MOFs but also delve into their metabolism within biological systems to augment the safety profile of nano-systems.

### 4.3. Silica-based nanoparticles

Mesoporous materials, particularly hollow mesoporous silica (HMS), have demonstrated invaluable value across various fields due to their unique combination of physical and chemical properties 97. These include ordered, customizable pore sizes (2~30 nm), substantial pore volumes (exceeding 1 cm³/g), expansive surface areas (surpassing 700 m²/g), and the presence of functional groups on their surfaces. Applications of HMS encompass the assembly of nanoparticles, catalysis, the design of drug delivery systems, the immobilization of biomolecules, and the adsorption and separation of gases and environmental pollutants. The high capacity of HMS for loading guest molecules, coupled with its ability to integrate catalytically active agents, endows it with a particular advantage in enhancing catalytic processes 98.

Silicon-based materials have also shown promise in gas storage, especially for H_2_ and natural gas 99. For instance, silane (SiH_4_) serves directly as a precursor for H_2_, releasing H_2_ upon decomposition; porous silicon, with its high specific surface area and porosity, is suitable for storing H_2_ and other small molecule gases; silicon-based nanostructures such as nanowires, nanotubes, and nanoparticles, as well as silicon-based adsorbents like silica gel and diatomite, capture gas molecules through physical adsorption; silicon-based MOFs utilize their porous structures, composed of metal ions and organic ligands, to store gases like H_2_ and CO_2_. Storage mechanisms of these materials include physical adsorption, chemical adsorption, and the formation of hydrides, all aimed at increasing storage capacity, improving kinetic performance, and reducing costs.

Despite the significant advantages of silicon-based materials in gas storage, there are relatively few reports in the literature on their direct use as gas carriers, possibly due to low loading or delivery efficiency. However, their potential as carriers for gas precursors has been demonstrated, as in the study by Gao *et al.*, where cuprous oxide (Cu_2_O) and a NO donor (BNN6) were encapsulated into dextran-modified mesoporous silica nanoparticles (MS), achieving H_2_S-triggered, NIR-controlled antibacterial and anti-cancer capabilities **(Figure 2C)** 100. With the advancement of nanotechnology and biomedicine, the application prospects of silicon-based materials in the field of gas therapy are broad. Although progression have been made, the development of new porous materials should be explored to further improve biocompatibility and biodegradability.

### 4.4. Two-dimensional materials-based gas delivery nanoplatforms

Two-dimensional (2D) materials, encompassing graphene, black phosphorus, transition metal dichalcogenides such as MoS_2_ and WS_2_, and 2D MOFs, have demonstrated remarkable potential in applications ranging from gas separation and sensing to gas transportation. These materials can form sub-nanometer sieving channels, facilitating precise molecular separation, particularly in gas separation applications. By integrating 2D materials as the scaffold for separation membranes and embedding ionic liquids into the 2D nanochannels, efficient gas separation can be achieved 101.

**Figure 3:**
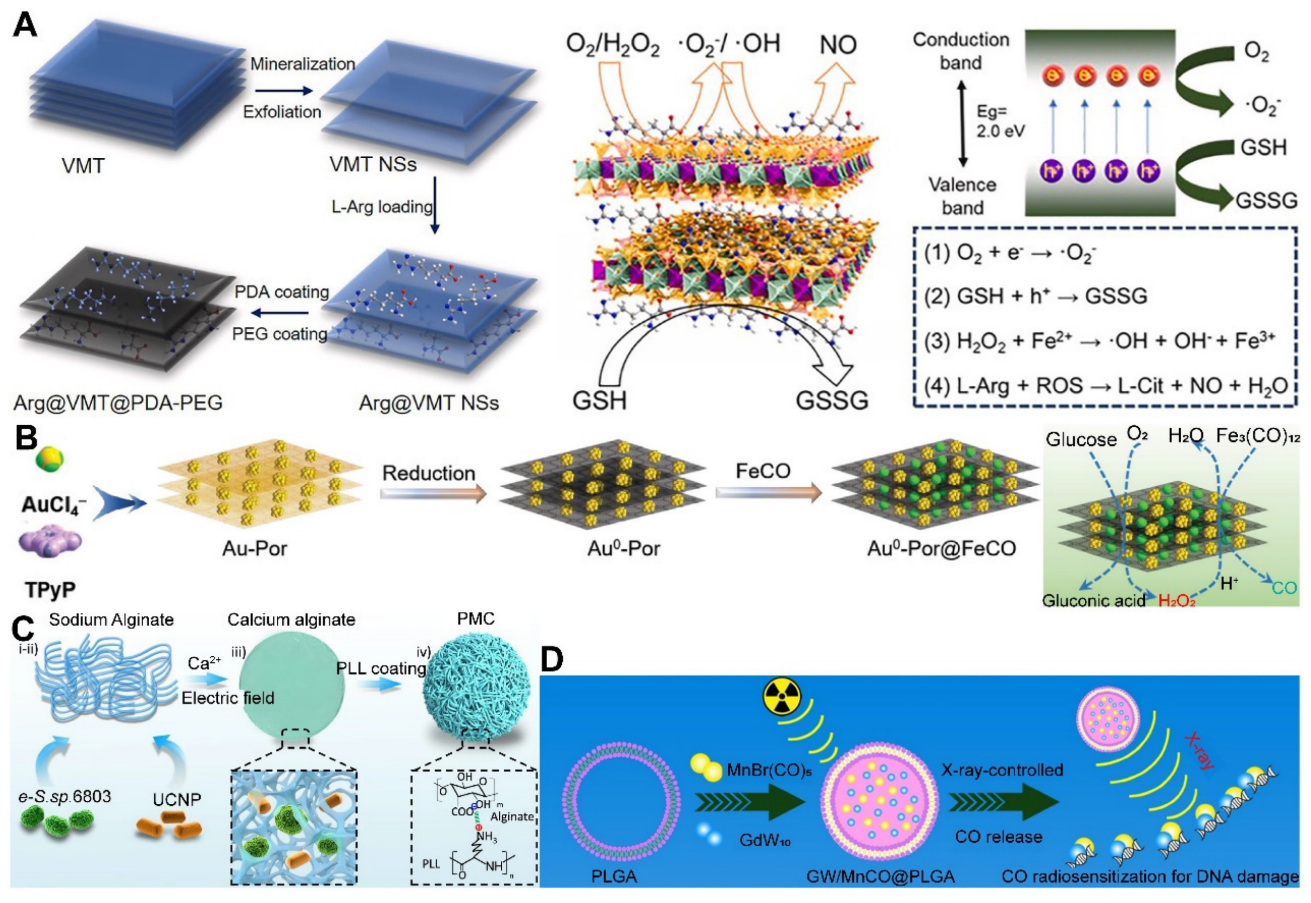
Gas delivery system using 2D material, photosynthetic cyanobacteria, and PLGA as gas carriers. (A) A 2D material-based gas delivery nanoplatforms (Arg@VMT@PDA-PEG NSs). Adapted with permission from 103, copyright 2023 Elsevier. (B) Another 2D material-based gas delivery nanoplatforms (Au^0^-Por nanosheets). Reproduced with permission from 105, copyright 2023 Wiley-VCH. (C) A gas delivery system using photosynthetic cyanobacteria. Reproduced with permission from 107, copyright 2022 Weili Wang *et al.* (D) A gas delivery system using PLGA (GW/MnCO@PLGA). Reproduced with permission from 108, copyright 2022 Bin Liu *et al*.

Moreover, 2D materials have shown unique advantages as drug carriers. Owing to their distinctive physicochemical properties, they can act as vehicles for stimulus-responsive gas release molecules, capable of releasing therapeutic gases in response to external physical stimuli or changes in the TME. The ultra-thin nature and large surface area of 2D materials offer possibilities for designing controlled gas-release systems. By modulating the interlayer spacing and surface functionalization of the materials, effective adsorption, storage, and on-demand release of gas molecules (or gas precursor) can be realized 102. For instance, Nie *et al.* developed a nano-catalyst platform based on 2D porous vermiculite nanosheets (VMT NSs), which could adsorb L-Arg (a NO donor) and prevent its leakage in the bloodstream through a polydopamine (PDA) and polyethylene glycol (PEG) coating, achieving a tumor-specific NO release therapeutic strategy **(Figure 3A)** 103.

Despite the recognized potential of 2D materials in drug delivery systems, their limitations in stability and biodegradability should not be overlooked. For example, black phosphorus, although more biodegradable and biocompatible than graphene 104, faces challenges in industrial application due to the complexity of its production process and low yield. Additionally, degradable materials like MOFs may release metal ions upon degradation, potentially reducing biosecurity. In this regard, Zhou *et al.* designed a gold-based porphyrinic coordination polymer nanosheet (Au^0^-Por) for the delivery of CO-releasing molecules (MnCO). The Au^0^-Por nanosheets exhibit glucose oxidase (GOx)-like catalytic activity, capable of catalyzing glucose to produce H_2_O_2_. The generated H_2_O_2_ further catalyzed the decomposition of the loaded CO-RMs, enabling the *in situ* generation of sufficient CO gas for gas therapy **(Figure 3B)** 105.

### 4.5. Photosynthetic cyanobacteria

The application of photosynthetic cyanobacteria in cancer therapy represents an innovative field of research, offering new possibilities through the generation of O_2_ via photosynthesis, which is instrumental in the treatment of cancer. These cyanobacteria are capable of producing O_2_ under irradiation with specific wavelengths of laser light, thereby ameliorating the hypoxic conditions within tumors and enhancing the efficacy of PDT.

Furthermore, synthetic biology techniques can be employed to engineer O_2_-producing microalgae, which can augment the photosensitization effect or sensitize radiotherapy, thereby improving therapeutic outcomes and preventing tumor recurrence and metastasis, as well as promoting wound healing 106. Wang *et al.* constructed a micro-oxygen factory, known as photosynthetic microcapsules (PMCs), designed to provide a sustained supply of O_2_. These microcapsules encapsulated cyanobacteria and up-conversion nanoparticles (UCNPs), enabling photosynthesis under NIR irradiation and supplying a continuous flow of oxygen to the tumor region, thereby inhibiting tumor growth and metastasis** (Figure 3C)** 107. These studies demonstrate the broad potential of photosynthetic cyanobacteria in oncology, emerging as a focal point in the recent exploration of bacterial therapies for cancer treatment. The integration of these microorganisms with advanced nanotechnologies and synthetic biology paves the way for novel therapeutic strategies that could revolutionize the field of cancer medicine.

### 4.6. Other carriers (PLGA, UCNPs, biomolecules, *et al.*)

PLGA, UCNPs, biomolecules, and polymers are nanocarrier systems not originally designed for the direct encapsulation of gases. They are typically utilized for the delivery of bioactive molecules such as pharmaceuticals, proteins, and nucleic acids. However, through specialized design and modification, these systems can be repurposed for the delivery of gaseous therapeutic agents.

PLGA, with its biodegradability and biocompatibility, offers a promising platform for the controlled release of gas precursor molecules, particularly in applications requiring sustained gas release to maintain therapeutic effects. By adjusting the chemical composition and structure of PLGA, precise regulation of gas release kinetics can be achieved 104. Liu *et al.* crafted a nanoparticle system based on GdW_10_ nanoparticles (GW) and MnBr(CO)_5_ encapsulated within a PLGA matrix, which was designed for CO RT. Under X-ray sensitization, these nanoparticles generated O_2_^-^•, triggering apoptosis in GW and facilitating the on-demand release of CO **(Figure 3D)** 108. Surface modifications, such as cationic surface functionalization, have also been employed to augment the loading efficiency of antigens onto PLGA nanoparticles, paving the way for customized combination therapies.

UCNPs are composed of an inorganic matrix doped with a luminescent center (e.g. Er^3+^, Ho^3+^, Tm^3+^) and a sensitizer (Yb^3+^ commonly used), along with a matrix that houses the dopant ions, including halides, oxides, sulfides, and sulfur oxides. UCNPs have become indispensable in disease diagnosis and treatment due to their proficient conversion of luminescence. In the context of gas generation strategies, exogenous photo-stimulation technology has garnered significant interest for its capacity to precisely control the spatial-temporal release of gases. As the development of UCNP-based gas delivery systems progresses, future research will focus on enhancing the luminous efficiency of UCNPs and exploring alternative excitation wavelengths, such as the NIR-II region, for their biomedical potential.

Biological molecules, such as proteins, peptides, and lipids, have practical applications in the delivery and storage of gases. For instance, in erythrocytes, hemoglobin is tasked with the transportation and delivery of oxygen to various parts of the body, and the conveyance of carbon dioxide back to the lungs for gas exchange. In muscle cells, myoglobin serves as an oxygen reservoir, providing a supply for muscle tissues during periods of high energy demand. Furthermore, biological molecules, including proteins and liposomes, can be utilized as carriers for drug delivery, facilitating the targeted transport of therapeutic gases or their precursors to specific tissues or cells. Among these, liposomes have been the most extensively utilized as drug delivery vehicles, which possess unique advantages in encapsulating and delivering gas precursor molecules. The bilayer structure of liposomes provides a stable environment for gas molecules and allows for surface modifications that enable targeting of specific cells or tissues 109. However, the stability of liposomes and the encapsulation efficiency of gas molecules still require further improvement.

Furthermore, research on polymers as gas carriers may focus on the development of multifunctional integrated systems capable of responding to external stimuli or changes in biomarkers, achieving intelligent control over gas release. Additionally, the biocompatibility and biodegradability of polymer carriers are important directions for future development. Effective encapsulation and controlled release of gases typically require the use of chemical methods or special material designs.

## 5. Stimuli-responsive gas delivery system

### 5.1. Exogenous stimuli-responsive gas delivery system

Exogenous stimulus-responsive gas delivery systems, modulated by specific external stimuli such as light/heat, ultrasound, irradiation, and magnetic fields, offer new possibilities for precision medicine. The necessity of these systems is predicated on their ability to precisely target and treat pathological regions, significantly enhancing therapeutic efficacy while minimizing adverse effects on normal tissues. The superiority of these systems is manifested in the following aspects: 1) they can respond to specific pathological environments, ensuring that therapeutic gases are released at the required tissues or cells, thereby reducing systemic side effects and improving the safety and tolerability of the treatment. 2) On-demand release is facilitated by controlling the timing and rate of gas release through external stimuli, according to the therapeutic needs. Additionally, personalized treatment protocols can be tailored to the specific medical condition and therapeutic requirements of the patient. The forthcoming sections will provide a detailed introduction to various exogenous stimulus-responsive gas delivery systems, exploring their working principles and application examples.

#### 5.1.1. Near-infrared laser-responsive gas delivery system

Among the myriads of external stimuli, light emerges as a particularly convenient and frequently employed trigger for the controlled delivery of gases. The majority of photo-responsive gas release prodrugs are designed to respond to UV-visible light. However, this comes with the downsides of limited tissue penetration depth and the potential for phototoxicity 110*.* In contrast, NIR light, with wavelengths ranging from 650 to 1100 nm, is popular due to its enhanced tissue penetration and reduced phototoxicity, positioning it as an attractive alternative for photo-responsive gas release in biomedical applications 111.

**Figure 4:**
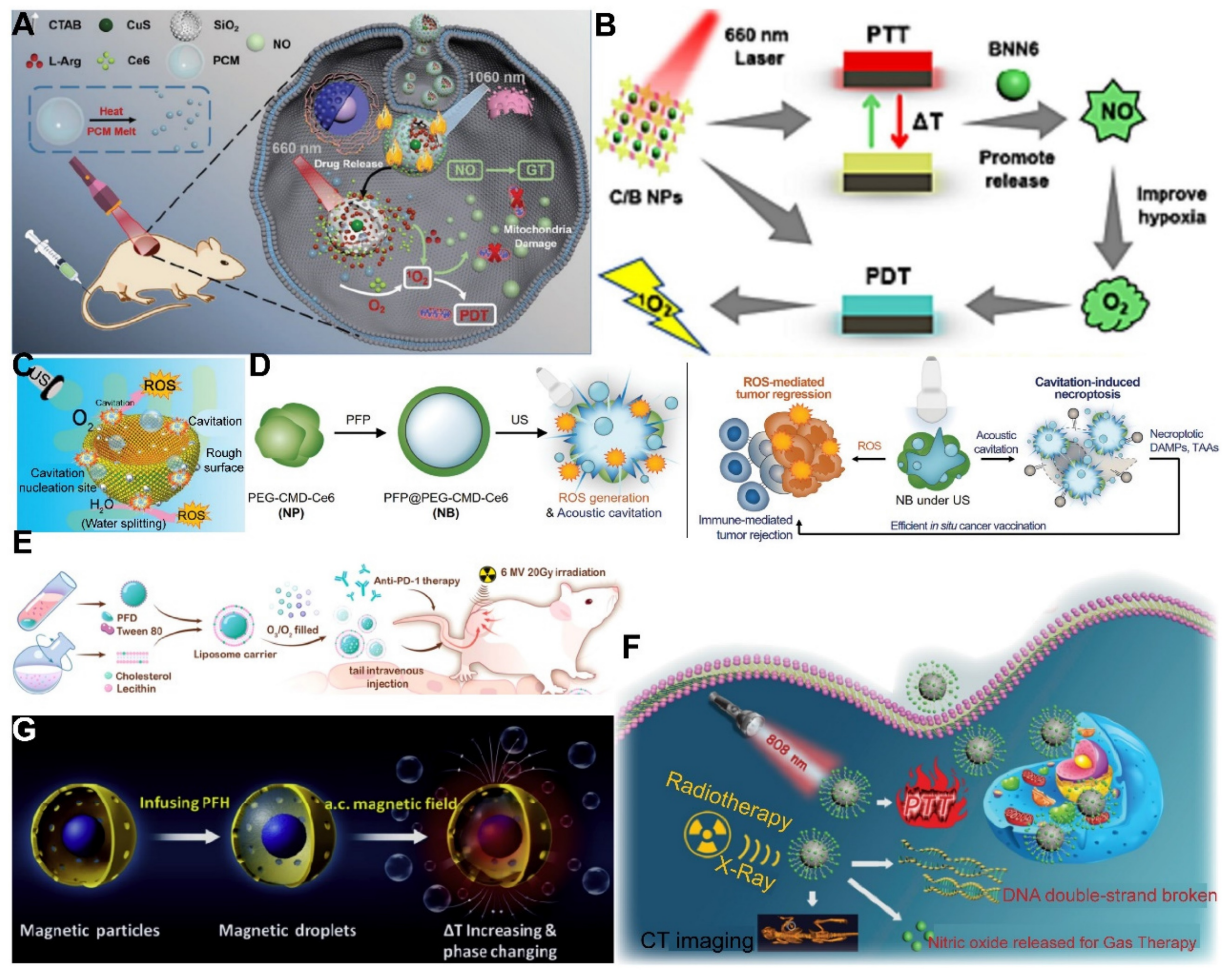
Exogenous stimuli-responsive gas delivery systems. (A) A NIR-responsive nanoplatform (CuS@SiO_2_-L-Arg@PCM-Ce6). Adapted with permission from 112, copyright 2021 Wiley-VCH. (B) A NIR-responsive nanoplatform (C/B@M NPs). Adapted with permission from 113, copyright 2024 Elsevier. (C) An US-responsive nanoplatform (APBN). Reproduced with permission from 115, copyright 2024 Xiahui Lin* et al.* (D) An US-responsive necroptosis-inducible NBs. Adapted with permission from 119, copyright 2020 Wiley-VCH. (E) An X-ray-responsive gas delivery system (O_3_/O_2__PFD@Liposome). Adapted with permission from 79, copyright 2022 Elsevier. (F) An X-ray-responsive gas delivery system (Bi-SNO NPs). Reproduced with permission from 124, copyright 2020 Royal Society of Chemistry. (G) A magnetic-responsive PFH-encapsulated MPs (MDs). Adapted with permission from 129, copyright 2017 Elsevier.

Utilizing NIR light for gas delivery, a thermally responsive multi-shell nanoparticle (CuS@SiO_2_-L-Arg@PCM-Ce6) was designed. Upon 1060 nm laser irradiation, the phase change material (PCM) within these nanoparticles transitions due to the photothermal effect of CuS, enabling the release of Ce6 and L-Arg. Ce6 generates ^1^O_2_ for PDT, and L-Arg, when oxidized, releases NO that targets mitochondrial and DNA integrity. The anti-cancer efficacy is monitored via fluorescence and NIR-II PA imaging of Ce6 and CuS **(Figure 4A)** 112. In an alternative approach, a core-shell nanoplatform (C/B@M) was developed with a pH/H_2_O_2_-responsive MnO_2_ coating that safeguards against premature ^1^O_2_ emission and mitigates tumor hypoxia, thus improving PDT. Upon 660 nm infrared exposure, the porphyrin-based COF core generates ^1^O_2_ and heat, while BNN6 decomposes to release NO, enhancing the hypoxic TME and enabling a synergistic multimodal treatment under MRI guidance **(Figure 4B)** 113. Light-responsive gas release systems harness the potential of non-invasive light sources to control gas release, which holds significant importance for enhancing therapeutic efficacy and minimizing side effects. Future research is likely to focus on improving the photostability of photosensitizers, optimizing the biocompatibility of nanocarriers, and developing multifunctional integrated systems capable of real-time monitoring and therapy under various imaging modalities.

#### 5.1.2. Ultrasound-responsive gas delivery system

The application of US for the controlled release of gas exhibits distinctive benefits, particularly in its capacity to target minute regions within human tissue and its ability to penetrate deeper tissue layers (1 MHz, 20 cm). This characteristic renders US highly effective for the treatment of tumors located in deeper tissues, a feat that light-based techniques often struggle to achieve 114. To bolster the efficacy of SDT, Lin *et al.* engineered an US-responsive nano-bomb, termed APBN, as an innovative sonosensitizer. APBNs facilitated the conversion of excess H_2_O_2_ within the TME into oxygen bubbles, addressing tumor hypoxia. Notably, the concave design of APBNs serves as an optimal site for O_2_ bubble aggregation, enhancing both their stability and proliferation. Evidence suggests that APBNs functioned as a bimodal contrast agent for US imaging (USI) and photoacoustic imaging (PAI), enhancing the precision of therapeutic interventions for deep-seated tumors** (Figure 4C)** 115.

Moreover, PFCs have garnered considerable interest in the realms of tumor imaging and treatment. Their appeal stems from their low boiling point and their propensity for phase change. PFCs have been the subject of research for improving extravascular US imaging and for intensifying the effects of high-intensity focused ultrasound (HIFU) therapy 116. Historically, the vaporization of PFC liquid was facilitated through acoustic droplet vaporization (ADV) technology, a process that initiates bubble formation within liquid nanodroplets, a result of the cavitation effect caused by ultrasonic pressure waves 117. In a recent development, optical droplet vaporization (ODV) has been introduced, employing laser irradiation to instigate the vaporization of PFC. Concurrently, magnetic droplet vaporization (MDV) has emerged as an alternative technique, leveraging a magnetic particle medium to generate heat and induce PFC vaporization 118.

PFCs, utilized as phase change materials in US-triggered release applications, offer innovative avenues for enhancing the contrast in US imaging and the efficacy of therapeutic interventions. Building on these principles, Wooram and colleagues have engineered a novel nanoplatform, which was composed of PFP, an amphiphilic polymer, and acoustic sensitizer Ce6. When subjected to US, PFP underwent vaporization, which led to the disruption of cell membranes and the release of DMAPs **(Figure 4D)** 119. However, the exploration of a variety of PFCs and phase change materials, along with their potential across different therapeutic modalities, remains a vast and uncharted territory.

#### 5.1.3. X-ray-responsive gas delivery system

X-ray irradiation stands as a prevalent clinical modality, distinguished by its low linear energy transfer (LET), facilitating profound tissue penetration without the need for invasive procedures 60. This form of radiation has demonstrated the potential to induce drug release at low dosages, independent of additional agents, thereby offering a controlled drug delivery mechanism 120. The profound penetration depth of X-ray radiation, coupled with its capacity for precise targeting and adjustable dosage parameters, makes it an ideal candidate for on-demand drug release. Furthermore, the integration of X-ray radiation with RT is recognized for its ability to augment therapeutic outcomes 121. Certain gas transmitters, including O_2_, NO, H_2_S, and O_3_, have been identified for their radio-sensitizing properties, which significantly elevate the sensitivity of hypoxic cells to X-ray radiation, thus enhancing RT efficacy 7. Moreover, the generation of ROS post-RT can impede DNA repair processes, presenting synergistic opportunities with PDT 122.

In the context of harnessing X-ray radiation for gas therapy, Zheng *et al.* introduced an innovative nano-system, O_3__PFD@liposome, which incorporated O_3_ within PFD liposomes. Upon exposure to X-ray radiation, this system catalyzed the production of a substantial number of •OH, thereby enhancing the efficiency of irradiation-induced neoantigen generation. *In vitro* analyses revealed that the yield of ROS from spontaneous decomposition of O_3_ was markedly lower compared to that stimulated by X-ray radiation, underscoring the efficacy of X-ray as a superior trigger for gas therapy in tumor elimination** (Figure 4E)** 79. While the O_3__PFD@liposome system can enhance the efficiency of neoantigen generation, the *in vivo* stability and targeting capabilities require further improvement to ensure the safety and efficacy of the treatment.

Nonetheless, the hypoxic nature of the TME presents a formidable challenge to RT, diminishing its anti-cancer potential. The inherent reduced absorption of X-rays by solid tumors necessitates higher radiation doses, which in turn poses a risk of collateral damage to adjacent healthy tissues. To counteract this, the targeted delivery of NO (1 µM~1 mM) to the tumor site has been proposed to sensitize RT by alleviating cancer cell hypoxia and promoting oxygen infiltration into the cancer cells 123. In this regard, Zhang *et al.* developed Bi-SH nanoparticles (Bi-SH NPs), which were further functionalized with S-nitroso mercaptan to facilitate the *in situ* release of NO under X-ray irradiation. The resulting nanoparticles demonstrated significant contrast in CT and NIR thermal imaging, thereby contributing to the enhanced efficiency of RT** (Figure 4F)** 124. Although Bi-SH NPs can respond to X-rays and release NO, a more in-depth investigation is needed regarding their distribution, metabolism, and potential long-term side effects within the body.

Several aspects should be addressed in the future. Firstly, developing more efficient X-ray-responsive materials to achieve faster and more controllable gas release. Secondly, enhancing the targeting of nanocarriers by surface modification or the use of activating ligands to increase their enrichment at tumor sites. Thirdly, exploring the combined use of various gas-delivering molecules to achieve superior therapeutic synergistic effects. And lastly, intensifying preclinical studies to assess the safety, efficacy, and long-term impact of these systems* in vivo*.

#### 5.1.4. Magnetic-responsive gas delivery system

Magnetic-responsive gas delivery systems have emerged as a promising avenue in cancer therapy, leveraging the unparalleled tissue penetration capabilities of electromagnetic energy. For instance, at a frequency of 500 kHz, an impressive 99% of the energy can penetrate through 15 cm of tissue, facilitating the conversion of this energy into heat by magnetic nanoparticles 125. This approach enables the targeted generation of heat for the destruction of cancer cells 126, the remote modulation of protein synthesis 127, the regulation of temperature-sensitive ion channels 128, and the vaporization of phase change contrast agents to enhance US imaging and thermal therapy.

In a notable study, Teng and his team developed microdroplets (MDs) for USI and tumor ablation by encapsulating PFH within magnetic mesoporous particles featuring hollow spaces. These MDs were adept at converting electromagnetic energy into heat, which was instrumental in tumor ablation. Concurrently, the vaporization of PFH resulted in the formation of bubbles, significantly improving USI capabilities **(Figure 4G)** 129. While MDs may require further enhancement of their stability and targeting capabilities to ensure the precision of therapy and minimize potential damage to surrounding healthy tissues. These advancements underscore the versatility and potential of magnetic-responsive gas delivery systems in the realm of cancer therapy. By harnessing the power of electromagnetic energy and the heat-generating capabilities of magnetic nanoparticles, these systems provide a multifaceted approach to cancer treatment, combining imaging, thermal therapy, and gas therapy in a cohesive and synergistic manner.

### 5.2. Endogenous stimuli-responsive gas delivery system

Endogenous stimulus-responsive gas delivery systems leverage naturally occurring signals or changes in conditions within the biological body to trigger the release of therapeutic gases. These systems are capable of synchronizing with the intrinsic physiological or pathological processes of the organism, thereby enabling more precise and personalized therapeutic regimens. Due to their responsiveness to endogenous stimuli, these systems exhibit higher compatibility with the biological system, reducing the potential for immune responses or side effects caused by exogenous substances. Their intelligent response mechanism aligns the release of gases more closely with therapeutic demands, such as releasing therapeutic gases in the hypoxic environment of tumor tissues, which minimizes the impact on normal cells and thus reduces systemic side effects. The subsequent content will delve into various endogenous stimulus-responsive gas delivery systems, including their design principles and operational mechanisms.

#### 5.2.1. Acid-responsive gas delivery system

The phenomenon of Warburg effect, characterized by a diminished glucose-6-phosphate dehydrogenase activity in cancer cells, results in an elevated glucose consumption without a concomitant increase in energy production 19. This metabolic anomaly leads to the accumulation of extracellular lactic acid and H^+^, thereby inducing acidosis, which is a hallmark of the TME 130. The extracellular pH of tumor tissues is significantly lowered to a range of 6.7 to 7.1, in stark contrast to the more neutral pH of approximately 7.4 observed in the surrounding normal tissues 131. This acidic milieu of tumor and inflammatory tissues provides a natural catalyst for the acid-responsive controlled release of therapeutic gases 132.

**Figure 5:**
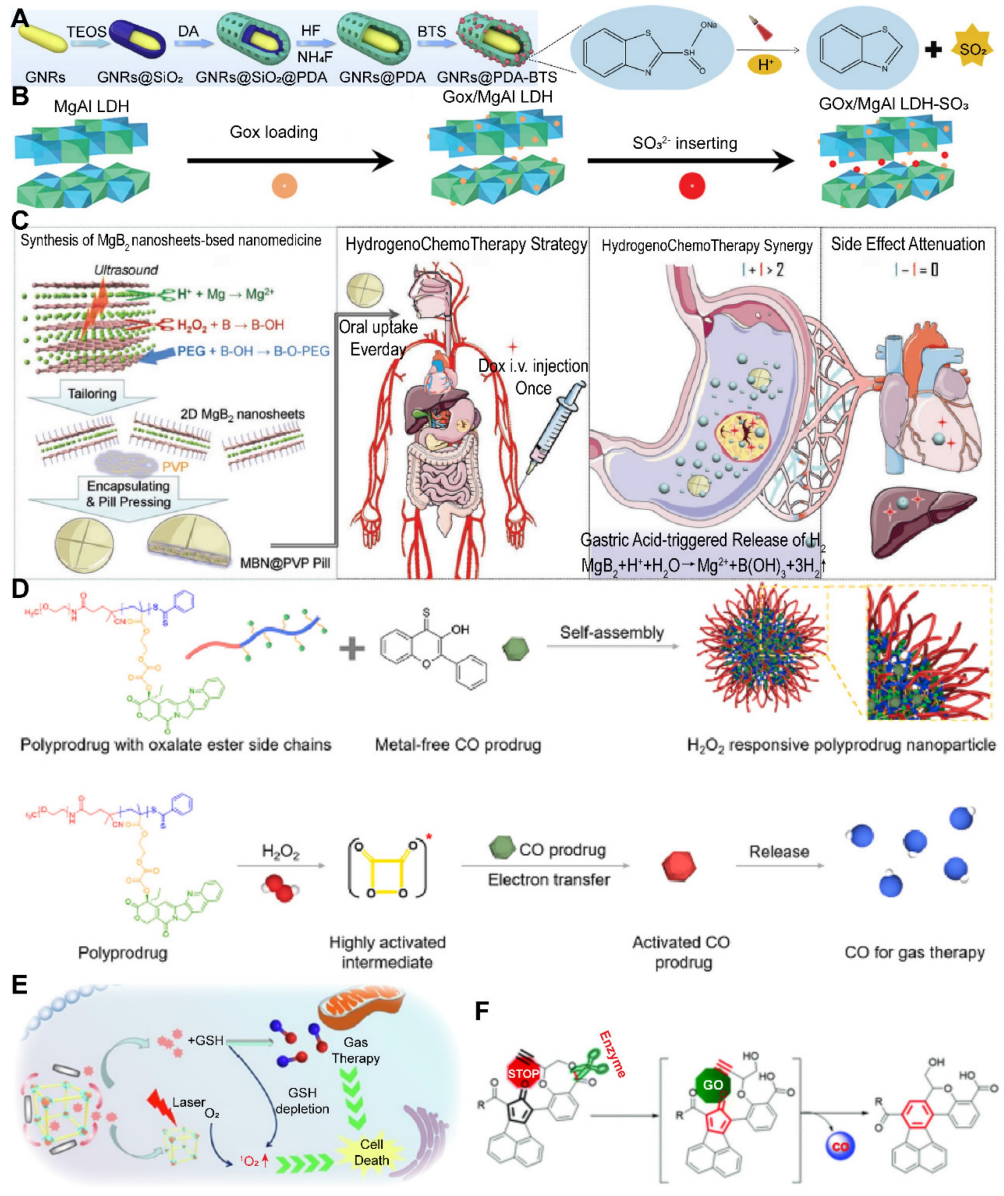
Endogenous stimuli-responsive gas delivery systems. (A) An acid-responsive SO_2_ delivery system (GNRs@PDA-BTS). Reproduced with permission from 133, copyright 2020 Elsevier. (B) An acid-responsive SO_2_ prodrug (GOx/MgAl-SO_3_ LDH). Reproduced with permission from 134, copyright 2021 Wiley-VCH. (C) An acid-responsive H_2_ delivery system (MBN@PVP pills). Reproduced with permission from 137, copyright 2019 Wiley-VCH. (D) H_2_O_2_-responsive CO-releasing molecules. Adapted with permission from 141, copyright 2024 Elsevier. (E) A GSH-responsive gas delivery system (Nic-MOF@HA). Reproduced with permission from 147, copyright 2022 Elsevier. (F) An enzyme-responsive CO prodrug. Reproduced with permission from 151, copyright 2017 Royal Society of Chemistry.

Despite the promise of gas therapy, the delivery and release of gases within biological systems remain constrained by limited penetration depths and the irreversible nature of endogenous stimuli, such as the GSH pathway. Addressing these challenges, Li *et al.* developed nano-gold rods coated with mesoporous dopamine (GPBRs) and doped with a SO_2_ prodrug, BTS. Findings reveal that the release of SO_2_ was significantly enhanced under acidic conditions (pH 5.0), with a 1.8-fold increase in relative fluorescence intensity compared to a neutral environment (pH 7.4). Furthermore, the release of SO_2_ was found to be accelerated under irradiation within the acidic TME **(Figure 5A)** 133. Sulfites are also a commonly used SO_2_ donor, and Chu *et al.* utilized them to construct a kind of nanosheets composed of Mg-Al layered dihydroxides (defined as MgAl-SO_3_ LDH). The glycolytic activity of GOx in this nano-system results in gluconic acid production, which in turn triggers SO_2_ release. This SO_2_, upon reacting with the excess intracellular H_2_O_2_, fosters ROS generation, inflicting substantial oxidative damage upon the cancer cells **(Figure 5B)** 134. However, the activity of glucose oxidase encapsulated in MgAl-SO_3_ LDH nanosheets may be susceptible to interference from other factors present in the TME, necessitating further research to optimize activity and stability of enzymes.

In the context of gastric cancer treatment, the highly acidic gastric microenvironment (pH ~ 1.2) presents a unique opportunity for the application of strong acid-responsive nanocarriers. Traditional H_2_ prodrugs, such as calcium hydride (CaH_2_) and magnesium hydride (MgH_2_), exhibit extreme instability in aqueous environments, particularly in the acidic milieu of the stomach, where they decompose spontaneously and release H_2_ at suboptimal rates 135. Moreover, the low solubility of H_2_ in water (1.6 ppm) curtails the therapeutic potential of oral H_2_-rich water therapies. In contrast, oral H_2_ prodrugs offer a more viable approach for the continuous and sustained delivery of high concentrations of H_2_ to digestive tumors 136. Capitalizing on this concept, Fan* et al.* synthesized a novel 2D magnesium boride (MgB_2_) nanosheet (MBN) that functions as an H_2_ prodrug. This innovative nanosheet was highly sensitive to acidic PBS and demonstrates the ability to release H_2_ in a controlled manner in response to the acidic gastric microenvironment for up to 72 hours **(Figure 5C)** 137. This targeted release mechanism holds significant promise for the treatment of gastric cancer, offering a more effective therapeutic strategy.

#### 5.2.2. H_2_O_2_-responsive gas delivery system

The elevated levels of H_2_O_2_ in cancer cells, ranging from 3 to 26 times higher than those in normal cells, have inspired researchers to utilize the endogenous H_2_O_2_ within the TME to stimulate the release of therapeutic gases 138. The overabundance of H_2_O_2_ in the TME can be effectively converted into molecular oxygen by natural biological enzymes, such as catalase, or by inorganic enzyme mimics, including Au-, Pt-, Mn-, and Fe-based nanomaterials. This conversion can ameliorate the hypoxic conditions within tumors, thereby enhancing the efficacy of various immunotherapies, SDT, PDT, and RT 12*.*

Manganese carbonyl (MnCO) represents a class of metal-based carbonyl compounds that can engage with the excessive H_2_O_2_ present in cancer cells, leading to the generation of •OH. This reaction further oxidizes the Mn center, which, in turn, competes with Mn to form coordination compounds, triggering the release of CO from the Mn site. The strategic release of CO results in its effective accumulation at the tumor site, thereby augmenting the therapeutic impact of CO while circumventing the risk of CO poisoning 139. Xu and his team constructed a novel CO therapeutic platform, which can be activated by endogenous H_2_O_2_ once internalized by cancer cells, achieving photo-induced excited-state intramolecular proton transfer (ESIPT) and spatiotemporally controllable CO release 140. Despite these advances, enhancing the drug loading capacity and delivery efficiency of MnCO remains a significant challenge in the optimization of CO treatment strategies. In addition, a small molecule CO prodrug that is not based on MnCO was synthesized by Wang and colleagues. They designed a poly-prodrug nanomedicine capable of rapidly dissociating under the influence of endogenous H_2_O_2_ present in tumors, leading to a swift release of CPT and the generation of a high-energy intermediate, dioxetanedione. This energetic intermediate can transfer energy to adjacent CO prodrugs through a process known as chemiexcitation, thereby activating the release of CO. Concurrently, CPT enhances the production of H_2_O_2_ within the tumor microenvironment, facilitating a cascade release of both CPT and CO **(Figure 5D)** 141. The development of novel CO-releasing prodrugs is equally crucial for advancing the therapeutic strategy.

#### 5.2.3. GSH-responsive gas delivery system

GSH, a tripeptide mercaptan composed of glutamic acid, cysteine, and glycine, is predominantly found in the cytoplasm. Notably, cancer cells exhibit a significantly higher concentration of GSH (2~10 mM) compared to their normal counterparts (2~10 μM) 142. This disparity presents an opportunity to engineer cancer cell-specific nanocarriers that exploit the elevated GSH levels for targeted therapeutic intervention. A variety of GSH-responsive chemical bonds, including disulfide, diselenide/disulfide, thioether/selenide/tellurium, mercaptan, and ferrocene, have been identified and integrated into the design of GSH-responsive nano-systems for therapeutic applications.

The therapeutic use of NO is often constrained by its gaseous nature and fleeting half-life 143. Precision cancer therapy, therefore, necessitates the targeted delivery of NO and the activation of its production in a tissue-specific manner. Certain NO donors, such as nitrates, can catalyze the GSH/GSSG redox reaction, generating NO while depleting intracellular GSH, which in turn fosters the production of additional ROS 144. Moreover, natural NO donors like L-arginine can be oxidized by ROS generated during PDT, leading to the in situ generation of high levels of reactive nitrogen species (RNS) for gas therapy, although with some depletion of ROS 145. Nicorandil (Nic), a GSH-activatable NO donor, stands out as reduction-sensitive rather than oxidation-sensitive, thereby not consuming the ROS generated during PDT. Furthermore, Nic can reduce GSH, minimizing ROS loss and significantly enhancing ROS production, which is instrumental in eliminating cancer cells. In a study of Xia *et al.*, Nic was encapsulated within porous porphyrin-MOF nanoparticles. Upon reaching the tumor site, Nic generated NO in response to high levels of intracellular GSH. This NO then reacted with superoxide radical anions to form highly cytotoxic ONOO^-^ molecules, thereby significantly boosting the efficacy of PDT 146 **(Figure 5E)** 147. In the future, by integrating advanced materials science with bioinformatics, more precise and efficient therapeutic strategies can be developed. For instance, leveraging machine learning algorithms to predict the specific levels of GSH within various TME, more accurately responsive nanocarriers can be designed to tailor to the unique characteristics of each cancer type.

#### 5.2.4. Enzyme-responsive gas delivery system

Enzymes, as fundamental regulators of cellular processes, often exhibit altered expression levels under various pathological conditions, such as cancer. These changes lead to an increase in enzyme levels, which provides the possibility for enzyme transformation and imaging activation of nanomaterials. Leveraging the enzymatic characteristics within the TME, intelligent nano systems can be designed to respond comprehensively to the activity of multiple enzymes. Such systems can provide a holistic overview of tumor anatomy, physiology, and molecular information through a single imaging nanoplatform, thereby enabling multimodal imaging 148.

For instance, the overexpression of matrix metalloproteinases (MMPs) throughout the stages of tumor progression allows for the design of MMP-cleavable peptide sequences, enabling the activation of nano-probes under the catalysis of MMPs and enhancing the imaging signal 149. Similarly, the expression of serine proteases in tumor tissues enables nanoplatforms to respond to their activity through specific peptide sequences, further enhancing the imaging signal 150. Additionally, enzymes with specific expression in tumors, such as urease, caspases, and alkaline phosphatase (ALP), can activate multimodal imaging, providing vital information for the diagnosis and treatment of cancer.

The design strategy for enzyme-responsive nanomaterials primarily involves the conjugation of imaging molecules with enzyme-cleavable linkers or the utilization of enzyme activity to trigger the decomposition or structural changes of nanomaterials, leading to the release or activation of imaging molecules. This design allows for the specific activation of nanomaterials within the TME, providing high-contrast tumor imaging that aids in the diagnosis and therapeutic monitoring of cancer. Enzyme-responsive chemical bonds, such as ester, amide, peptide, phosphoester, disulfide, alkyne, and epoxy bonds, typically involve specific chemical structures that undergo changes under the action of particular enzymes, resulting in drug release or the generation of active molecules. For example, the esterase-activated CO prodrugs mentioned by Wang and his team are “held” by an esterase-sensitive cleavable linker with alkyne groups, preventing cyclo-addition reactions with the cyclopentadienone moiety until the constraint is released. In the presence of esterases, these prodrugs can effectively release CO, while in their absence, the half-life of CO release is significantly prolonged. This strategy not only enhances the specificity and sensitivity of imaging but also provides new approaches for cancer therapy **(Figure 5F)** 151.

## 6. Application of gas therapy in cancer imaging and therapy

### 6.1. Application of gases in cancer imaging

Medical imaging stands as a cornerstone scientific discipline, enabling the visualization of the human body's internal tissues and organs. The purpose is achieved by elucidating their morphological structures, densities, and functional properties. The field operates on the principle of interaction between different forms of energy or media and the human anatomy, which includes X-rays, electromagnetic fields, US waves, radionuclides, and other modalities.

Among the various applications of medical imaging, the use of gas molecules is particularly significant. These molecules are employed to enhance the contrast of images, thereby refining the accuracy and clarity of diagnostic assessments. The domain of medical imaging is expansive and is traditionally categorized based on the underlying types of information carriers utilized. This categorization encompasses a spectrum of modalities, including X-ray imaging, nuclear medicine imaging, MRI, USI, optical photography, NIR imaging, PAI, and microwave imaging. Each of these imaging modalities brings distinct advantages to the table and is calibrated to meet specific diagnostic requirements, offering a comprehensive and multifaceted strategy for the visualization and diagnosis of the human body.

#### 6.1.1. Photoacoustic imaging

PAI emerges as an innovative, non-invasive, and non-ionizing biomedical imaging technique that capitalizes on the expansion and contraction of light-absorbing materials induced by pulsed lasers to elicit US signals 152. By merging the high optical contrast of tissue imaging with the profound penetration capabilities of USI, PAI is proficient at capturing tissue images with exceptional resolution and contrast 153. This method circumvents the limitations imposed by light scattering and surpasses the depth constraints of high-resolution optical imaging, which is typically confined to approximately 1 mm, enabling deep tissue imaging up to 50 mm *in vivo*. The attributes of PAI—encompassing its non-invasive nature, high sensitivity, specificity, favorable signal-to-background ratio, and superior spatial and temporal resolution—position it as a valuable adjunct to MRI and CT. While endogenous chromophores such as Hb, oxyhemoglobin, melanin, and fluorescent proteins serve as natural contrast agents for PAI, the development of exogenous contrast agents has garnered significant interest, particularly for diseases like breast cancer and glioma, which lack sufficient endogenous contrast 152.

**Figure 6:**
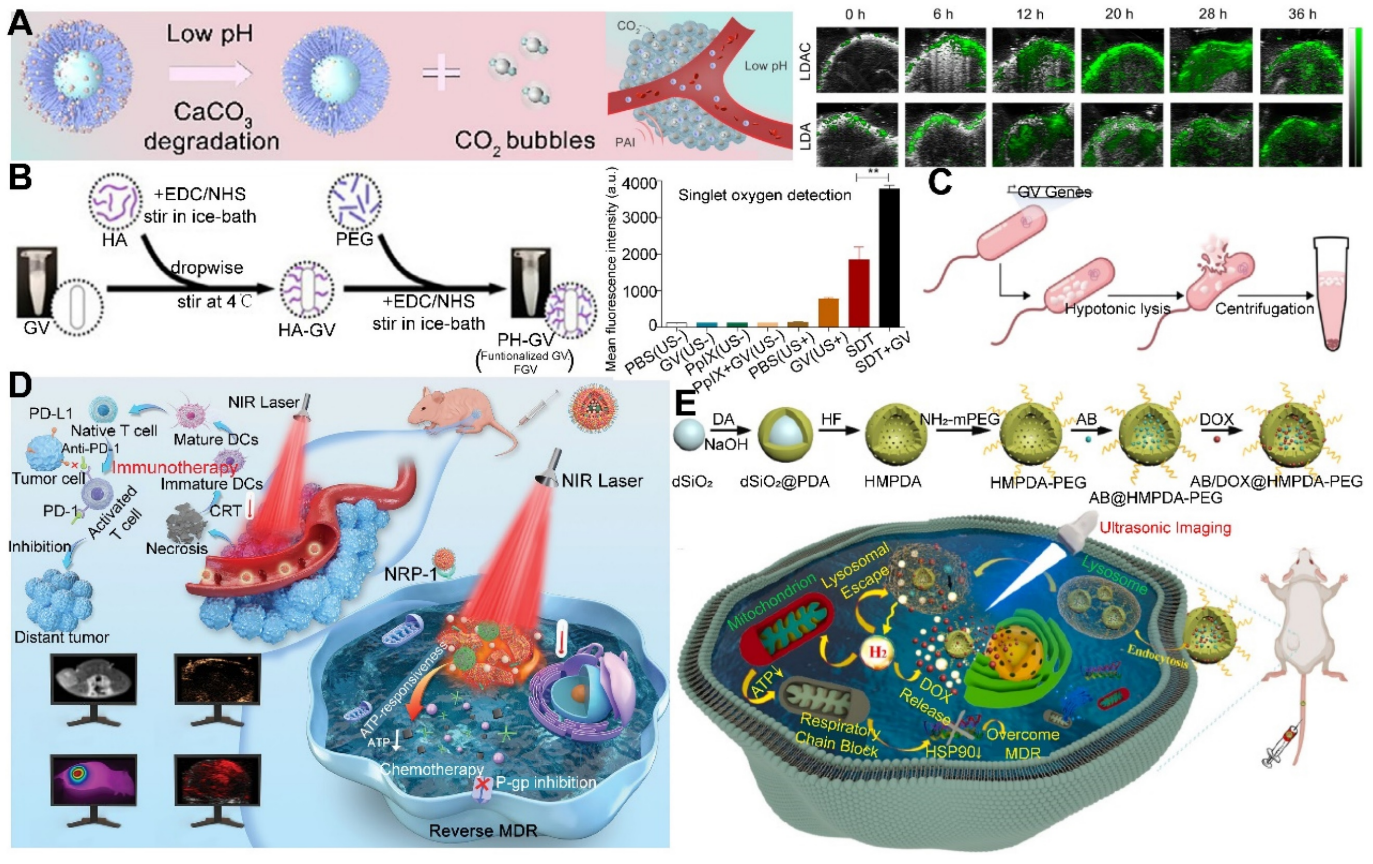
Application of gas therapy in cancer imaging. (A) A nanoplatform (LDAC NPs) used for PAI/ NIR-II FI dual-mode imaging. Adapted with permission from 155, copyright 2023 Elsevier. (B) A nanoplatform designed to achieve SDT and ultrasonic imaging (GVs). Reproduced with permission from 159, copyright 2021 Elsevier. (C) Improved GVs with stable ultrasonic contrast signal. Reproduced with permission from 160, copyright 2022 Mingjie Wei *et al.* (D) A nanoplatform designed for ultrasonography imaging, PAI, and MRI (P@TF NPs). Reproduced with permission from 164, copyright 2023 Wiley-VCH. (E) A nanoplatform AB/DOX@HMPDA-PEG for improving ultrasonic imaging efficacy. Reproduced with permission from 166, copyright 2021 Elsevier.

CO_2_-based contrast agents absorb light energy at specific wavelengths, elevating local temperatures and causing pressure fluctuations due to thermal expansion, which are detectable by acoustic detectors as photoacoustic signals 154. Xu and co-workers engineered LDAC NPs, which encapsulates lanthanide-doped nanoparticles, gold nanoparticles (Au NPs), and a coating of calcium carbonate (CaCO_3_). Upon irradiation with a 980 nm laser, these LDAC NPs initiated a robust up/down-conversion process that yields intense fluorescence within the NIR-II window. Concurrently, they transfer energy to the associated Au NPs through a fluorescence resonance energy transfer (FRET) mechanism, which in turn facilitates the generation of a PAI signal. Moreover, the CaCO_3_ layer undergoes degradation within the acidic milieu of the TME, leading to the formation of CO_2_ microbubbles. These microbubbles augment the PA signal via a cavitation effect, thereby enhancing the overall imaging contrast **(Figure 6A)** 155. Subsequent research endeavors may extend to the development of novel nanomaterials capable of being activated within the NIR-II window, endowed with tunable switchable properties, thereby facilitating more precise imaging. The specificity and sensitivity of PAI imaging are also anticipated to be enhanced. Concurrently, by integrating multimodal imaging techniques, such as combining PAI with MRI or CT and other imaging modalities, a more comprehensive set of disease information can be provided, aiding in more accurate diagnostics and therapeutic monitoring.

#### 6.1.2. Ultrasound imaging

USI, recognized for its cost-effective equipment and non-invasive nature, is extensively employed in clinical settings to procure cross-sectional images of organs 156. This technique allows for the observation of organ movements and rapid imaging without causing discomfort or posing any inherent risks. Often complemented by SDT, USI can induce the generation of deleterious free radicals, leading to cellular damage and apoptosis. While USI boasts swift image acquisition and timely diagnosis, its contrast and spatial resolution do not match the superior quality offered by CT and MRI modalities. Nonetheless, the sensitivity and precision of USI can be augmented through the application of contrast agents, such as microbubbles, which create cavitation effects during ultrasonic activation, thereby enhancing image quality 157.

Gas vesicles (GVs), nanobubbles filled with gas and enclosed by protein shells, are produced by marine bacteria and archaea, including cyanobacteria 158. These structures can serve as US imaging contrasts akin to microbubbles 156. Song *et al.* engineered GVs with surface modifications for the specific targeting of CD44^+^ cells, leading to preferential accumulation at tumor sites and achieving a synergistic effect in SDT and USI **(Figure 6B)** 159. However, traditional GVs are relatively small, and their imaging contrast is suboptimal, particularly for *in vivo* applications. Addressing this limitation, Wei *et al.* isolated GVs from Halobacteria NRC-1 in a rugby ball shape, which demonstrated a robust and stable ultrasonic contrast signal in mouse liver tumors, with a signal strength 6.84 times greater than that of lipid microbubbles** (Figure 6C)** 160.

While traditional micro-bubbles (MBs) enhance US echo signals by resonance at diagnostic frequencies 161, their large particle size (typically several microns) restricts their application to blood pool imaging, as only nanoparticles smaller than 700 nm can effectively penetrate the leaky vasculature of tumors 162. Additionally, MBs are prone to clearance by the reticuloendothelial system (RES), resulting in reduced blood circulation times and suboptimal tumor accumulation. On the other hand, MBs in the 1~100 nm nanoparticle range contribute minimally to USI due to a marked reduction in nonlinear backscattering.

To enhance the extravasation of microbubbles while maintaining strong echo signals and improving imaging contrast, two strategic approaches have been devised: (1) the coupling of nanodroplet-to-droplet evaporation techniques, and (2) *in situ* gas generation through chemical reactions. In the first approach, PFCs, due to their low boiling points, are increasingly valued for their ability to permeate nanosized containers and produce bubbles and radiant forces upon external triggering, thus serving as phase change contrast agents 163. The unique properties of phase change contrast agents have garnered increased interest in tumor imaging and therapy, including extravascular US imaging and enhanced HIFU therapy 116. Cheng *et al.* engineered a thermo-responsive nano-system, denoted as tP@TFP NPs, which, upon 808 nm laser irradiation, harnessed the significant photothermal properties of TA-Fe^3+^ to convert light energy into heat. This process facilitates the phase transition of PFP and subsequent rupture via ODV, thereby releasing P@TF NPs encapsulated within the liposomal structure. Concurrently, a multistage reaction is triggered, which can be monitored in real-time using ultrasonography imaging, PAI, and MRI **(Figure 6D)** 164. For the second strategy, a controlled gas release platform is essential, as uncontrolled gas release may precipitate embolism and unpredictable side effects 165. Wang *et al.* introduced AB/DOX@HMPDA-PEG nanoparticles, containing ammonia borane (AB) and DOX, which are acid-sensitive and release H_2_ and DOX, respectively. This design improves USI efficacy, facilitates the lysosomal escape of nanoparticles, and blocks the mitochondrial respiratory chain, achieving synergistic tumor imaging and therapy **(Figure 6E)** 166. However, the stability of gas vesicles or microbubbles is crucial for their circulation within the body and therapeutic efficacy. Further research is warranted to enhance the biostability and half-life of these carriers.

#### 6.1.3. Magnetic resonance imaging

MRI is a medical imaging technique that leverages the principles of nuclear magnetic resonance (NMR) 16. This technology primarily relies on the magnetic resonance phenomenon of protons within a strong magnetic field, reconstructing images of the internal structures of the human body by detecting the signals they emit. As a non-invasive measurement technique, MRI can capture internal structural images of various substances. It is capable of producing high-contrast, clear images without the need for ionizing radiation or contrast agents 167. The use of gases in MRI is a relatively new research domain, particularly in the area of pulmonary imaging 168. Traditional MRI faces challenges in imaging the lungs, as they are primarily air-filled structures with a proton density significantly lower than other tissues, making imaging difficult 169. To overcome this challenge, scientists have explored the use of hyperpolarized gases as tracers for pulmonary imaging. Hyperpolarized gas MRI technology utilizes inert gases, such as Xenon (Xe) and fluorinated gases, which can dissolve in the lungs and produce distinct magnetic resonance signals, thereby enabling non-destructive, quantitative, and precise assessment of lung ventilation and gas exchange functions.

Xe is an inert gas that is non-toxic and harmless to the human body, exhibiting excellent biochemical inertness, lipid solubility, and chemical shift sensitivity. It can dissolve in pulmonary blood and tissues, generating varied magnetic resonance signals that facilitate lung imaging and can be utilized for early screening and therapeutic evaluation of lung diseases. In the field of oncology, Xe-enhanced MRI can be employed for the early diagnosis of cancer and for assessing the ventilation and gas exchange functions of normal lung tissue during treatment. This allows for the prediction of treatment side effects and the protection of healthy lung tissue. Additionally, Xe is used in hyperpolarized 129Xe gas MRI imaging techniques, which, in the study of lung cancer radiotherapy applications, can quantify the dosimetric impact on high-functioning lung regions, thus optimizing radiotherapy plans 170.

Helium is typically used in conjunction with Argon in a technique known as “Argon-Helium cryosurgery”, a method for the cryoablation treatment of cancer. This technique uses the rapid freezing and thawing effects of Argon and Helium gases to destroy cancer cells 171. Argon-Helium cryosurgery can be applied to the treatment of various solid tumors, including lung, liver, breast, and renal cancers, characterized by minimally invasive, painless, and rapid recovery features. The role of Helium in cryosurgery is to rapidly increase temperature after Argon freezing, achieving quick thermal therapy, which thoroughly destroys pathological tissues through the cold-heat reversal therapy. Cryosurgery can reduce tumor burden, improve patients' quality of life and survival time, and is particularly suitable for patients with advanced stages who cannot undergo surgical resection or those unwilling to endure the side effects of radio-chemotherapy.

In summary, Xe is primarily applied in the field of pulmonary imaging, especially in radiation-free pulmonary MRI, aiding in the early diagnosis and therapeutic assessment of cancer. Helium, often combined with Argon, has a broad application in cryoablation treatment for cancer, offering a minimally invasive and effective therapeutic option for solid tumors. However, there are some limitations: 1) Techniques such as Xe-enhanced ventilation CT (XeCT) and hyperpolarized 129Xe gas MRI may be costly and have limited availability; 2) In hyperpolarized ^129^Xe gas MRI, gas flow effects may significantly impact image quality, introducing additional noise and artifacts, and reducing the signal-to-noise ratio of the image 172; 3) The requirement for patients to inhale gases like Xe and hold their breath may be challenging for some patients, particularly those with respiratory difficulties or other pulmonary diseases. 4) Although hyperpolarized 129Xe gas MRI can provide detailed information about pulmonary ventilation and gas exchange, it may still need to be compared and integrated with traditional imaging techniques such as CT or PET-CT to determine its actual advantages in cancer assessment 173. 5) Some hyperpolarized gas MRI techniques may still be in the research and development stage and have not yet been widely applied in clinical practice. 6) The heterogeneity of tumors can affect the interpretation of imaging results, necessitating a comprehensive assessment of tumor characteristics in conjunction with other clinical information and imaging technologies.

### 6.2. Combined administration of gas therapy for cancer treatment

To optimize synergistic therapeutic effects and tailor treatment regimens, the strategic integration of gas therapy with other therapeutic modalities is essential. Gas therapy, particularly NO therapy, is often applied in conjunction with other treatment modalities due to its multifaceted benefits. The anti-cancer effects of NO can augment the efficacy of chemotherapy or radiotherapy while reducing dosages to minimize side effects. Moreover, gas therapy contributes to overcoming drug resistance in cancer cells by modulating the redox state of the TME, thereby enhancing the penetration and efficacy of other therapeutics. Additionally, the integration of multiple treatment approaches addresses the heterogeneity of tumors, leading to a more comprehensive therapeutic outcome. Gas therapy can also activate or enhance the immune response, improving the immune system's recognition and clearance of tumors when used in combination with immunotherapy. As an emerging treatment strategy, the incorporation of gas therapy offers novel avenues and research directions for cancer treatment, thereby enhancing therapeutic efficacy, reducing adverse effects, and expanding treatment options for patients.

#### 6.2.1. Synergistic gas therapy and starvation therapy

**Figure 7:**
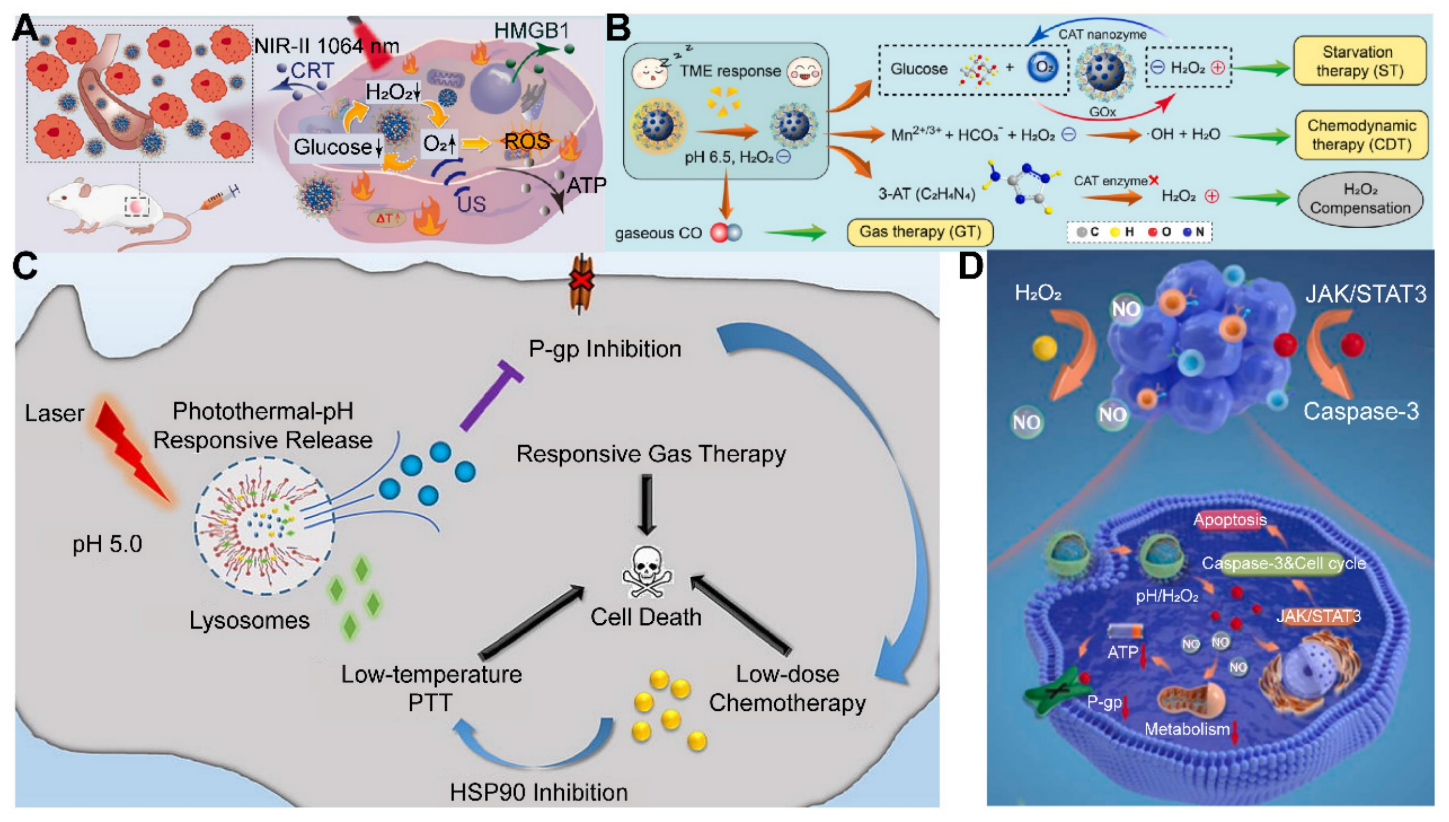
Application of gas therapy in cancer treatment synergy with starvation therapy and chemotherapy. (A) Synergistic PDT, SDT and gas therapy (PGI). Reproduced with permission from 175, copyright 2022 Elsevier. (B) Synergistic cancer starvation therapy, chemotherapy and gas therapy (PGMA Nus). Adapted with permission from 177, copyright 2022 Elsevier. (C) Synergistic chemotherapy and gas therapy (IGN Lipo). Reproduced with permission from 179, copyright 2023 Elsevier. (D) Synergistic chemotherapy and gas therapy to overcome MDR (F-L-O@M NPs). Reproduced with permission from 182, copyright 2023 Elsevier.

Over the past decades, considerable attention has been directed towards drug interventions that target cancer metabolism 174. One such approach is starvation therapy, which seeks to enhance cancer treatment by depleting exogenous glucose via the enzymatic action of GOx, thereby obstructing the energy supply to the tumor site. However, the glucose-consuming reaction mediated by GOx is oxygen-dependent, presenting a challenge in the hypoxic TME that must be addressed. To overcome this, various O_2_ self-supplying nanoplatforms have been designed. Zhou *et al.* integrated a nano-catalyst PdPt with GOx and the acoustic sensitizing agent IR780 to create PdPt@GOx/IR780 (PGI). This construct facilitates the conversion of intracellular H_2_O_2_ to O_2_, providing ample substrates for GOx-mediated glucose consumption within the tumor, leading to energy and nutrient deprivation and subsequent tumor starvation **(Figure 7A)** 175.

An inherent limitation of targeting cellular metabolic pathways is the inability to completely inhibit the energy supply to tumors by glucose depletion alone, as alternative energy sources like lactic acid, glutamine, and pyruvate can still fuel cancer cell proliferation 176. Oxidative phosphorylation (OXPHOS), a downstream process in cellular energy metabolism, can be disrupted by CO, which impairs the mitochondrial electron transport chain (ETC) and halts ATP production. However, the non-specific release of CO in normal tissues can reduce its concentration in cancer cells, leading to metabolic and redox adaptations and potentially drug resistance. A platinum (Pt) nano-urchin-based platform integrated GOx, MnCO, and 3-AT (3-amino-1,2,4-triazole, a catalase inhibitor) was designed by Huang* et al*. In the TME, MnCO decomposed to emit CO and release 3-AT and Mn^2+/3+^. The glucose depletion by GOx, along with CO, impaired mitochondria and cellular metabolism. Crucially, 3-AT suppressed catalase, increased intracellular H_2_O_2_ to amplify cascade reactions, thereby enhancing the synergistic therapy of gas therapy and starvation therapy **(Figure 7B)** 177. While some solutions have been proposed in the literature, the development of efficient and specific CO release platforms continues to be a significant challenge. Research can focus on the precise temporal and spatial control of gas release to synchronize with the effects of starvation therapy.

#### 6.2.2. Synergistic gas therapy and chemotherapy

Chemotherapy remains a prevalent strategy in the oncological area, celebrated for its rapid and pronounced therapeutic effects 178. Nonetheless, the efficacy of chemotherapy is often compromised by the adverse effects associated with the inert components of the delivery systems and the suboptimal drug loading efficiency. The inherent aggressiveness of tumors and the development of acquired drug resistance, coupled with the necessity to use low concentrations due to high cytotoxicity, lead to the current limitations in the efficacy of chemotherapy.

To enhance the efficacy of chemotherapy, innovative strategies have been proposed. A notable example is a self-assembling nano-missile reported by Wang *et al.*, which was loaded with IR-780, glycyrrhizic acid (GA), and a NO donor, diethylenetriamin (DETA) NONOate. Upon cancer cell uptake, the nano-missile facilitates low-temperature PTT and releases NO and GA. The released NO further enhances therapy by inhibiting P-glycoprotein (P-gp), reversing drug resistance and bolstering chemotherapeutic effects of GA **(Figure 7C)** 179. Additionally, the side effects on normal tissues and the multidrug resistance (MDR) phenotype of cancer cells present significant challenges for the clinical application of chemotherapy. Cancer cells can develop MDR through a variety of mechanisms, including reduced drug uptake, increased intracellular drug efflux, diminished intracellular drug concentration, activation of DNA repair mechanisms, upregulation of metabolic pathways, and stimulation of detoxification processes 180. Numerous studies have highlighted the potential of NO in reversing MDR by inhibiting DNA repair, hypoxia-inducing factors, and transporter activity, among other mechanisms. However, the high reactivity of NO and its donors results in poor *in vitro* and *in vivo* stability and low bioavailability, which complicates the direct targeting of cancer cells.

To surmount these challenges, strategies have been executed to inhibit ATP-binding cassette (ABC) transporter overexpression and suppress cellular respiration to impede the energy supply essential for ABC transporter function 181. For example, Chen *et al.* developed ferulic acid-L-arginine nanoparticles (F-L NPs) that not only induce cancer cell apoptosis by targeting the JAK/STAT3 pathway but also produce NO, which impairs mitochondrial function and reduces ATP levels. This dual action down-regulates P-gp expression, enhancing the apoptotic effect and the overall therapeutic outcome **(Figure 7D)** 182. These examples illustrate the potential of combining gas therapy with chemotherapy to overcome drug resistance and enhance treatment efficacy.

#### 6.2.3. Synergistic gas therapy and radiotherapy

RT, a non-invasive treatment modality, utilizes X/γ-ray radiation to target malignant tissues with precision, guided by imaging techniques to minimize the radiation exposure to surrounding healthy tissues. However, the presence of significant hypoxia in solid tumors can attenuate the effectiveness of RT 183. Additionally, certain cancer subtypes and cancer stem cells exhibit inherent resistance to X-ray radiation, necessitating the development of radiosensitizers to enhance the sensitivity of anoxic or radioresistant tumors to X-ray irradiation 184.

**Figure 8:**
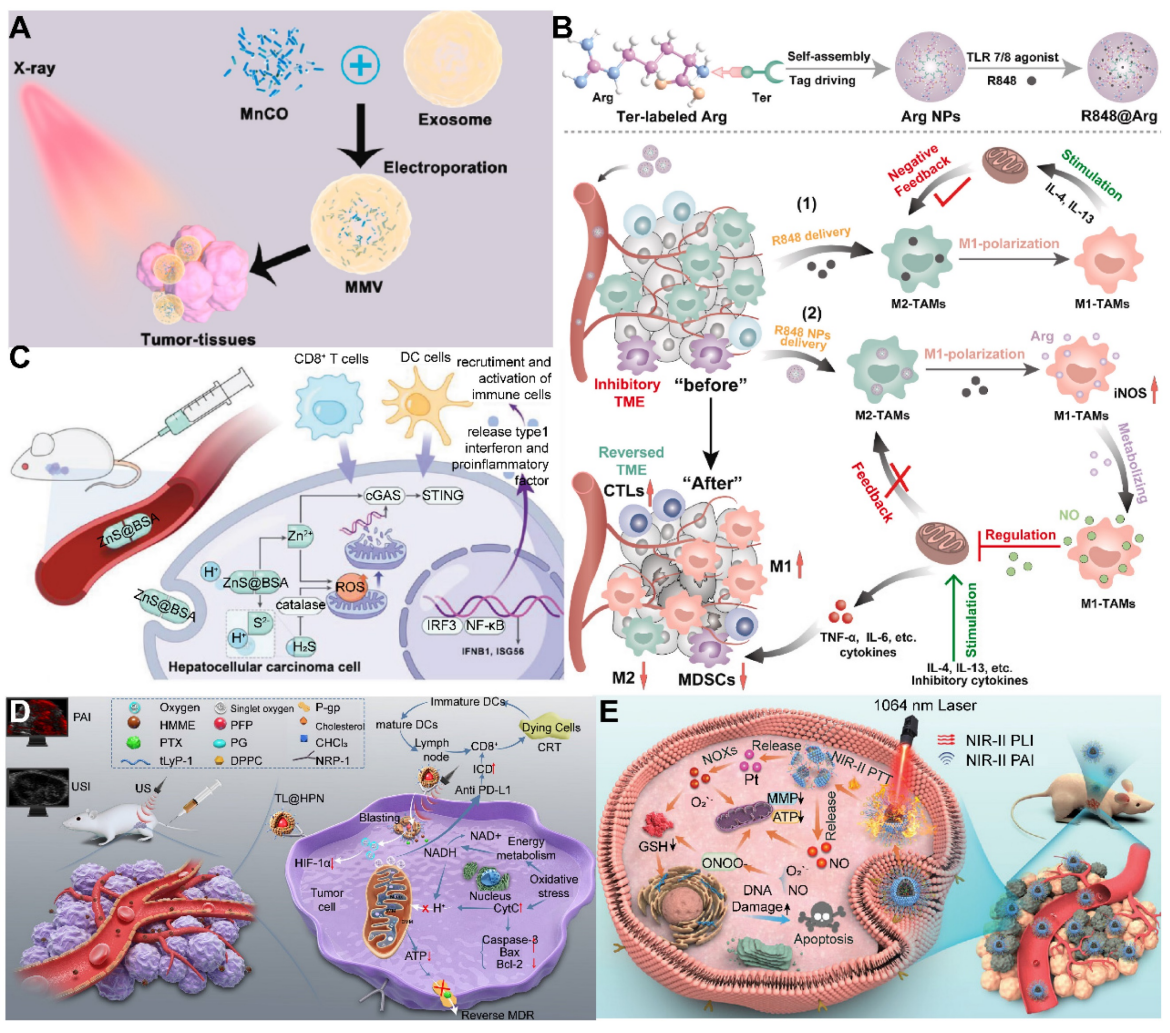
Application of gas therapy in cancer treatment synergy with RT, immunotherapy, SDT, PDT and PTT. (A) Synergistic RT and gas therapy (MMV). Adapted with permission from 185, copyright 2021 Elsevier. (B) Synergistic gas therapy and immunotherapy (R848@Arg). Adapted with permission from 190, copyright 2024 Xiaqing Wu* et al*. (C) Synergistic gas therapy and immunotherapy targeting cGAS/STING pathway (ZnS@BSA). Reproduced with permission from 193, copyright 2021 Wiley-VCH. (D) Synergistic gas therapy and SDT (TL@HPN). Reproduced with permission from 197, copyright 2022 Xun Guo *et al*. (E) Synergistic gas therapy and PTT (PBT/NO/Pt). Reproduced with permission from 200, copyright 2024 Mi Zhang *et al.*

Gas transmitters, such as O_3_, O_2_, NO, and H_2_S, have been identified for their potential as radiosensitizers, enabling a synergistic approach that combines RT with gas therapy. Zhu *et al.* developed a mixed nanoplatform, termed MMV, which consists of tumor-derived exosomes (EXOs) loaded with MnCO to facilitate targeted low-dose irradiation. This platform demonstrated a remarkable therapeutic effect under a very low dose of irradiation (2 Gy), outperforming the conventional high-dose (6 Gy) RT **(Figure 8A)** 185. The synergistic strategy of gas therapy combined with RT, while promising, has certain limitations that require refinement: the hypoxic environment within solid tumors impairs the efficacy of RT, necessitating the development of more effective oxygenation strategies to enhance the RT efficiency in hypoxic regions. Additionally, increasing the tumor specificity of radiosensitizers is crucial for improving the safety of the treatment.

#### 6.2.4. Synergistic gas therapy and immunotherapy

In the realm of cancer treatment, immunotherapy has witnessed significant advancements by leveraging the host's immune system to combat malignancies 186. In this context, gas molecules are frequently employed as adjuvants in combination with other therapeutic strategies to exert tangible anti-cancer effects. It is therefore critical to devise approaches that stimulate anti-tumor immunity through the applications of biologically active gas molecules. For instance, elevated levels of NO can activate M1 tumor-associated macrophages (TAMs), significantly curbing tumor metastasis and enhancing the efficacy of immunotherapy 187. Concurrently, H_2_S therapy has been shown to diminish the immunosuppressive activity of myeloid-derived suppressor cells (MDSCs), reinvigorating T cell proliferation and bolstering melanoma immunotherapy 188. Additionally, CO can modulate the biological pathways of cancer cells and macrophages by inducing mitochondrial ROS.

In the TME, TAMs constitute over half of the tumor volume and play a dual role: the M1 phenotype can eliminate cancer cells by generating pro-inflammatory cytokines and recruiting T cells, while the M2 phenotype fosters cancer cell growth by producing cytokines and inducing T regulatory cells. Notably, the M1 phenotype is characterized by high levels of inducible nitric oxide synthase (iNOS), which catalyzes the conversion of L-arginine to NO 189. Based on this concept, Zheng* et al.* developed an arginine-based nano-assembly, R848@Arg, incorporating a toll-like receptor 7/8 agonist, resiquimod (R848), and arginine served as a NO donor. R848 facilitated the phenotypic transition of TAMs from the M2 to the M1 phenotype, thereby enhancing the antitumor immune response within the TME, and NO produced by arginine prevented the negative feedback polarization of TAMs. The nanoparticles collectively enhanced tumor suppression efficacy, effectively reducing tumor proliferation and the presence of MDSCs **(Figure 8B)** 190. Furthermore, CO has been shown to regulate TAM polarization in the TME. Low concentrations of CO (100 ppm) can increase the number of M1 TAMs, activating T cells and natural killer (NK) cells for anti-cancer immunity, while higher concentrations (250 ppm) can enhance the infiltration of M2 TAMs, contributing to immunosuppression 191.

Another approach to bolster innate immunity involves the activation of cyclic guanosine adenosine monophosphate (GMP-AMP) synthetase/interferon gene stimulator (cGAS/STING) pathways. cGAS detects damaged DNA and activates STING, leading to the up-regulation of type I interferon (IFN) and pro-inflammatory cytokines, thereby inhibiting tumor progression 192. Based on this premise, Cen and colleagues developed ZnS@BSA nanoclusters to generate H_2_S under acidic conditions, which led to ROS accumulation in cancer cells and subsequent mitochondrial damage and DNA release, thus activating cGAS/STING signals. The intracellular Zn^2+^ further enhances the activity of cGAS catalytic enzymes, amplifying the cGAS/STING signaling pathway and inducing greater immune cell infiltration to eliminate cancer cells **(Figure 8C)** 193. Leveraging the bioactivity of gas molecules to activate or modulate the immune system, the potential of gases such as NO, H_2_S, and CO have been witnessed in fostering anti-cancer immune responses. However, several challenges and limitations persist. For instance, the acidic conditions and immunosuppressive characteristics of the TME pose challenges to the efficacy of gas therapy. Moreover, the synergistic effects of gas therapy with other immunotherapies, such as immune checkpoint inhibitors, warrant further investigation to assess the impact of combined treatment strategies.

#### 6.2.5. Synergistic gas therapy and sonodynamic therapy

Although SDT and PDT share similar mechanisms, SDT has emerged with several distinct advantages 194. US, the driving force behind SDT, is characterized by its robust tissue penetration, reaching several centimeters deep with minimal attenuation, allowing for the precise targeting of deep-seated tumors by modulating US frequency and intensity 195. Despite these advantages, preclinical studies have shown that the therapeutic efficacy of SDT alone is suboptimal.

To enhance the effectiveness of SDT, gas-assisted strategies have emerged as a promising approach. These strategies primarily involve the formation of MBs to facilitate deep tissue penetration and rapid drug release. Additionally, these strategies leverage US-mediated gas cavitation to induce cell necrosis, with the subsequent lysis of cell membranes releasing TAAs that can trigger ICD. The released gas can also serve as a contrast agent for US imaging, enabling the integration of cancer diagnosis and treatment.

The utilization of MBs in SDT has garnered considerable research interest. MBs, composed of a biocompatible shell surrounding a gas core, offer an ideal structure for the delivery of therapeutic gases 196. Upon US activation, the gases within the core are released, augmenting the local therapeutic effect in SDT. Oxygen-carrying MBs, for instance, have been extensively explored to enhance tumor oxygenation and improve the outcomes of RT and PDT. Guo *et al.* developed a US-controlled nano-system (TL@HPN), which employed PFP as the core to adsorb O_2_ and was decorated with a targeted peptide (CGNKRTR) attached to a liposome. This construct incorporates porphyrin monomethyl ether (HMME) and paclitaxel (PTX). Upon US stimulation, of drugs and O_2_ were triggered to release, amplifying the generation of intra-tumoral ROS to induce mitochondrial apoptosis and maximize ICD **(Figure 8D)** 197. However, the efficacy of SDT alone is relatively low, necessitating the combination with other therapeutic modalities to enhance the anti-cancer efficacy.

#### 6.2.6. Synergistic gas therapy and photodynamic/photothermal therapy

PDT has been in clinical use for over three decades and has emerged as a promising cancer treatment modality, distinguished by its minimally invasive nature, low toxicity and side effects, high selectivity, and the preservation of facial features and vital organ functions 198. PDT diverges fundamentally from conventional laser surgery, relying on a series of photochemical reactions rather than direct physical intervention. The clinical implementation of PDT involves the administration of a photosensitizer to the patient, followed by the application of laser light to the targeted lesion area. PHOTOFRIN® and Hipophen are among the widely utilized photosensitizers in contemporary clinical practice.

However, the hypoxic TME poses a significant challenge to the efficacy of PDT, given that O_2_ is an essential substrate for the photosensitive reaction. To address this, a variety of O_2_-supplying nano-systems have been engineered to alleviate hypoxia. Zhang *et al.* designed a sustained O_2_ supply system based on cerium nanoparticles and hydrogel (GHCAC), which infiltrated water to react with CaO_2_, generating a continuous flow of O_2_ through the catalase-like activity of HCePA. This system demonstrated the capability to effectively alleviate tumor hypoxia for up to seven days, thereby enhancing the therapeutic outcomes of PDT. Additionally, the ^1^O_2_ generated during PDT could oxidize L-Arg, leading to the generation of NO and further reacting with ROS to form highly toxic RNS, such as nitrous oxide and peroxynitrite 199. A range of NO prodrugs has been developed, including organic nitrates/nitrites, metal-NO complexes, nitrosamines, and S-nitroso mercaptan. Building upon this foundational concept, Sun *et al.* developed a novel multifunctional nanogenerator, PBT/NO/Pt, capable of sensing the acidic pH of the TME responsively releasing NO and ROS culminating in the *in situ* ONOO^-^generation. The nanogenerator, upon excitation with NIR-II region light, facilitates high-resolution fluorescence and PAI of deep-seated tumor tissues, and is endowed with an efficient photothermal conversion capability, positioning it as a viable candidate for PTT **(Figure 8E)** 200. Future strategies are primarily focused on optimizing the design of photosensitizers and gas precursors to enhance the selectivity and accumulation of nano-systems within tumor tissues, while concurrently improving the efficiency of photothermal conversion and the stability of gas concentrations.

## 7. Conclusions and outlooks

The field of gas therapy, with its potential to revolutionize cancer treatment, has seen a surge in interest. A variety of gases, including CO, H_2_S, and NO, are known to modulate cellular processes, while O_2_ and O_3_ have demonstrated the ability to induce cell death through free radical production. The recent inclusion of H_2_ and SO_2_ has further expanded the therapeutic options available within this domain.

Despite the promising nature of these findings, challenges inherent to the application of gas therapy in cancer treatment can not be underestimated. A critical challenge lies in the precise control of gas concentration and delivery, which is paramount for therapeutic efficacy. The determination of optimal dosage levels for the various gases involved is a complex task, complicated by the fact that the impact of these gases can vary significantly based on the type and stage of cancer. This variability underscores the need for a nuanced understanding of the molecular and biological mechanisms that underlie the anti-cancer effects of these gas molecules.

To address these challenges, the development of gas-specific delivery systems is crucial. These systems must be tailored to the unique physical and chemical properties of each gas to ensure the safe and effective delivery of therapeutic agents. The design of gas-releasing vectors that respond to specific stimuli within the TME represents a significant step forward. However, the instability of these carriers within the harsh TME environment can lead to uncontrolled gas release, necessitating the development of more targeted and secure delivery methods.

While gas therapy is a promising modality, it is not typically used as a standalone treatment. Instead, it is often employed in conjunction with other therapeutic approaches, such as chemotherapy, PDT, RT, and immunotherapy, to enhance treatment outcomes. The integration of gas therapy with real-time imaging techniques, including US imaging, also holds potential for the simultaneous diagnosis and treatment of tumors.

The translation of nano-gas generators into clinical practice requires overcoming several hurdles. The preparation of these systems must be both repeatable and efficient, and their long-term biosafety, biodistribution, excretion, and compatibility with biological systems must be thoroughly evaluated before clinical trials can be initiated. Achieving high standards in these areas will be essential for the successful clinical translation of gas therapy.

Looking ahead, gas therapy presents itself as an emerging “green” strategy with the potential for on-demand release, offering a promising avenue for personalized medicine. The applications may extend beyond oncology, and with ongoing research and development, gas therapy may well establish itself as a fundamental component of the multidisciplinary approach to cancer treatment.

## Figures and Tables

**Table 1:** Key concentration parameters and cancer therapeutic mechanisms of gases.

Gas	Cancer therapeutic concentration	Blood poisoning concentration	Anti-cancer mechanisms
O_2_	Most experiments use the μM to mM range as the tumor hypoxia fraction, with a range of 10% to 30%.	O_2_ toxicity is concentration- and time-dependent: 1 to 21 hours of exposure to 100% pure O_2_; or 2 to 24 hours of inhalation of 60% to 100% O_2_; or more than 24 hours of inhalation of 40% to 60% O_2_.	Inhibit cancer cell growth or metastasis by alleviating hypoxia.
NO	Several to hundreds of μmol.	In acute exposure, inhaling high concentrations of NO (for example, over 150 ppm); or long-term exposure to lower concentrations of NO (for example, a few ppm to several tens of ppm).	Activate the apoptotic pathway, inhibit tumor angiogenesis; damage cellular biofunction after being converted into peroxynitrite.
CO	Several tens of ppm.	Inhaling more than 35 ppm, or a carboxyhemoglobin (COHb) level greater than 10%.	Inhibit the activity of the mitochondrial respiratory chain by suppressing cytochrome c oxidase, causing intracellular energy metabolism disorders, while also affecting apoptosis and immune responses through signaling pathways similar to those of NO.
H_2_S	Several μM to several tens of μM.	Inhaling more than 10 ppm.	Regulate the redox balance within cells, activate or inhibit various signaling pathways, affecting apoptosis, proliferation, and angiogenesis.
SO_2_	Acting at the mM level, but the minimum effective concentration is still unclear.	Inhaling more than 0.5 ppm.	Affect the redox status inside cells, intervene with cell cycle and apoptotic pathways, and possibly inhibit angiogenesis.
H_2_	1% to 4%.	Generally does not cause poisoning.	Neutralize harmful ROS through its selective antioxidant properties, reduce oxidative stress, regulate cellular signal transduction, and suppress inflammatory responses.
O_3_	Usually related to triggering conditions in research articles.	Inhaling more than 100 micrograms per cubic meter (μg/m³).	Directly damage the cancer cell membrane with its strong oxidizing property, increase intracellular ROS, induce oxidative stress, leading to DNA damage and apoptosis.

**Table 2:** Selected physical properties of frequently used PFCs 83.

Compound	C_5_F_12_	C_6_F_14_	C_8_F_18_	C_8_F_17_Br	C_10_F_18_	N(C_3_F_7_)_3_	N(C_4_F_9_)_3_
Boiling point (°C)	28~30	58~60	99~106	143	140~143	131	178
O_2_ solub. (vol%, 25 °C)	About 54	70	52.1	52.7	40.3	45.3	33.2~38
CO_2_ solub. (vol%, 25 °C)	-	156	-	210	142	166	127~152

Solub., abbreviation as solubility. C_5_F_12_, perfluoropentane. C_6_F_14_, perfluorohexane. C_8_F_18_, perfluorooctane. C_8_F_17_Br, perfluorooctylbromide. C_10_F_18_, perfluorodecalin (cis + trans). N(C_3_F_7_)_3_, perfluorotripropylamine. N(C_4_F_9_)_3_, perfluorotributylamine.
